# Development of LC-FAIMS-MS and its application to lipidomics study of *Acinetobacter baumannii* infection

**DOI:** 10.1016/j.jlr.2024.100668

**Published:** 2024-10-10

**Authors:** Jianjun Li, Jacek Stupak, Arsalan S. Haqqani, Greg Harris, Hongyan Zhou, Sam Williamson, Rui Chen, H. Howard Xu, Wangxue Chen

**Affiliations:** 1 Human Health Therapeutics Research Centre, National Research Council Canada, Ottawa, Ontario, Canada; 2 Department of Biological Sciences, California State University Los Angeles, Los Angeles, CA, USA

**Keywords:** FAIMS, lipidomics, mass spectrometry, phospholipid, NPLC, *Acinetobacter baumannii*, bacterial infection

## Abstract

The recent advances in mass spectrometry (MS) technologies have enabled comprehensive lipid profiling in biological samples. However, the robustness and efficiency of MS-based lipidomics is compromised by the complexity of biological samples. High-field asymmetric waveform ion mobility spectrometry (FAIMS) is a technology that can continuously transmit one type of ion, independent of the mass-to-charge ratio. Here we present the development and application of LC-FAIMS-MS/MS-based platform for untargeted lipidomics. We used 3 optimally balanced compensation voltages, i.e., 29 V, 34 V and 39 V, to analyze all subclasses of glycerophospholipids. The reproducibility of the method was evaluated using reference standards. The reproducibility of retention times ranged from 0.9% to 1.5% RSD; whereas RSD values of 5%–10% were observed for peak areas. More importantly, the coupling of a FAIMS device can significantly improve the robustness and efficiency. We exploited this NPLC-FAIMS-HRMS to analyze the serum lipid profiles in mice infected intranasally with *Acinetobacter baumannii*. The temporal profiles of serum lipids after *A. baumannii* inoculation were obtained for 4 h, 8 h, and 24 h. We found that nearly all ether PC and ether PE lipids were significantly decreased 8 h after inoculation. The resultant volcano plot illustrated the distribution of 28 increased and 28 decreased lipid species in mouse sera 24 h after inoculation. We also found that a single ether PE composition can comprise multiple isomeric structures, and the relative abundance of each isomer could be quantified using the newly developed NPLC-FAIMS-PRM method. We have demonstrated that the proposed LC-FAIMS-MS is a valuable platform for lipidomics.

## Graphical Abstract

**Figure:**
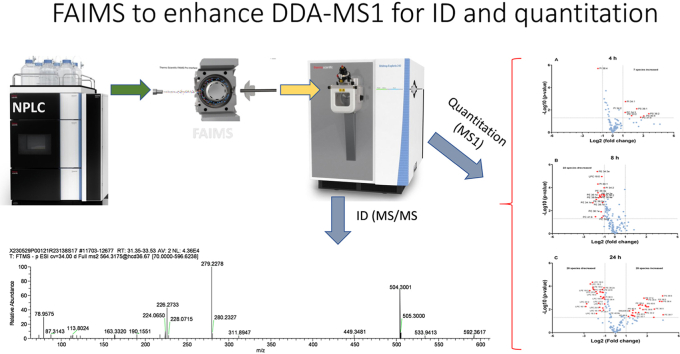

Lipidomics is an emerging discipline that studies lipids of biological systems on a large scale, involving the pathways and networks of cellular lipids in biological systems (1, 2). It plays an essential role in defining the biochemical mechanisms of lipid-related disease processes, including infectious diseases, through the identification and quantification of thousands of lipid molecular species. Based on LIPID MAPS, lipids can be classified into eight categories, including fatty acyls, glycerolipids, glycerophospholipids, sphingolipids, sterol lipids, prenol lipids, and saccharolipids (3, 4, 5). Glycerophospholipids are membrane structural constituents and key players in cell signaling, homeostasis, and inflammatory and immune responses. The general glycerophospholipid structure includes three main components: a glycerol backbone, a functionalized phosphate ester group, and fatty acyl (or alkyl ether) chains (3, 4). The phosphate moiety is esterified at the *sn*-3 position of the glycerol backbone and is also coupled to a polar functional group or headgroup. Lysophospholipids (LPLs) are phospholipids with only one acyl chain at either the *sn*-1 or the *sn*-2 position of the glycerol backbone, depending on the product formed by the action of phospholipidases. Phospholipidase A1 catalyzes the cleavage of fatty acids in position 1 of phospholipids; while phospholipidase A2 catalyzes the cleavage of fatty acids in position 2 of phospholipids. The inventory of lipid molecules found in blood plasma (plasma lipidome) offers insights into individual metabolism and physiology in health and disease (6).

Mass spectrometry (MS) has proven to be a powerful tool for both quantitative and qualitative analysis of biomolecules (1, 7, 8, 9). The development of state-of-the-art high-resolution mass spectrometry (HRMS) instruments, e.g., Orbitrap or time-of-flight (TOF), and in combination with different types of high-performance liquid chromatography platforms, including hydrophilic interaction (HILIC) and reverse-phase liquid chromatography (RPLC), has greatly advanced lipidomics research. HILIC enables the separation of lipid classes based on headgroup composition and use ESI-compatible mobile phases to improve ionization efficiency and reproducibility (7, 8). On the other hand, a well-optimized RPLC method can separate lipids with different fatty acyl chains (10). Furthermore, this separation mechanism makes it easier to identify phospholipids in untargeted lipidomic studies. The advanced high-resolution accurate-mass Quadrupole-Orbitrap mass spectrometers are synonymous with high sensitivity and resolving power. It delivers the depth of analysis to the lowest levels with high quantitative accuracy and precision for proteomics research. However, the ruggedness and robustness of MS remain challenging for lipidomics applications, especially for a multi-omics MS facility environment, in which the nature of lipidomics samples can be an unavoidable source of cross-contamination for proteomics experiments. The coupling of high-field asymmetric waveform ion mobility spectrometry (FAIMS) to Orbitrap mass spectrometer has shown great potential in lipidomics applications by removing the chemical background and preventing MS interface contamination (11, 12, 13, 14, 15, 16, 17). FAIMS device can be easily removed and installed without breaking the mass spectrometer vacuum.

*Acinetobacter baumannii* is a Gram-negative bacterial pathogen commonly transmitted in healthcare (hospital and community) settings, causing up to 2% of all healthcare-associated infections in Canada (∼5,000 cases per year) and the USA (∼50,000 cases per year), with more than 1 million cases per year globally (18). *A. baumannii* infections cause different clinical syndromes, including pneumonia, septicemia, urinary tract infections, wound and surgical site infections, and meningitis (18). Mortality can range from 5% in general wards up to >50% in intensive care units (19). The prevalence of multidrug-resistant *A. baumannii* isolates has rapidly increased over the last decade, and it is now one of the most common multidrug-resistant pathogens in clinical settings (20, 21). Many *A. baumannii* clinical isolates are intrinsically resistant to penicillin, chloramphenicol, aminoglycosides, and several other classes of antibiotics (sulfonamides, macrolides). *A baumannii* can develop resistance via either lipid A modification (e.g., with phosphoethanolamine) (22, 23) or even lipopolysaccharide loss in the outer membrane (24, 25). Carbapenems (imipenem, meropenem or doripenem) are now the last-resort treatments for this pathogen (18). The prevalence, high mortality, and pervasive development of resistance have made *A. baumannii* one of the most important emerging pathogens globally and a Priority 1 (Critical) bacterial pathogen in the WHO priority pathogens list for R&D of new antibiotics (26). In a 2019 global survey of 204 countries and territories, *A. baumannii* infection is the fifth leading cause of bacterial infections and accounts for nearly 0.5 million deaths during this period (21). In addition, 2019 to 2022 data from the CDC shows that there was an alarming increase (78%) in carbapenem-resistant *Acinetobacter* infections at the beginning of the COVID-19 pandemic (27). Lipids can act as important inflammatory mediators during the infection process. For example, changes in lipid components of the serum or plasma can occur during sepsis and bacterial infection (28, 29, 30). Previous studies have revealed a decrease in most fatty acid species in plasma following infection with *A. baumannii* (31). Another feature of *A. baumannii* is the preferential scavenging of exogenous lipids, including host-derived polyunsaturated fatty acids (PUFAs), for membrane phospholipid synthesis (32).

In this article, we report on the development of LC-FAIMS-MS/MS techniques for lipidomics studies and their application to study lipid changes in mouse sera after *A. baumannii* infection. The results demonstrate that the combination of HILIC-based normal phase liquid chromatography (NPLC) and HRMS, coupled with a FAIMS device, offers a powerful qualitative and quantitative workflow for serum lipid profiling.

## Materials and methods

### Chemicals and materials

HPLC-grade water, methanol (MeOH), isopropanol (IPA), and hexane were obtained from Thermo Fisher Scientific. Formic acid, ammonium hydroxide, and methyl tert-butyl ether (MTBE) were purchased from Sigma-Aldrich. Lipid standards were from Avanti Polar Lipids. A Standard Vortex Mixer from VWR Scientific was used for sample mixing.

### Animal study

Eight- to ten-week-old, specific-pathogen-free female BALB/c mice were obtained from Charles River Laboratories (St. Constant QC). We used female mice for Acinetobacter infection to avoid littermates, fighting each other and for ease of handling by research staff. Although some groups have reported higher susceptibility of female mice to this infection (33), we and others did not find overt differences in the clinical signs, body weight changes, mortality or tissue bacterial burdens between male and female mice (34, 35). The animals were housed and used in accordance with the Canadian Council on Animal Care Guide to the Care and Use of Experimental Animals, and all experimental procedures were approved by the institutional animal care committee.

For intranasal inoculation, *A. baumannii* LAC-4, a hypervirulent strain previously described by us (34), was streaked from frozen stock onto a brain heart infusion (BHI, MilliporeSigma) plate and incubated overnight at 37°C. Bacterial cells were then harvested into tryptic soy broth (TSB, MilliporeSigma) and incubated at 37 °C for 2–3 h with 200 rpm shaking to an OD_600_ of 0.85. Bacterial cells were then centrifuged and resuspended in 0.85% saline at the desired inoculation concentration, based on a pre-determined OD_600_ value. Mice were anesthetized by isoflurane inhalation, then inoculated intranasally with 5 × 10^7^ CFU *A baumannii* in 50 μl. The actual inoculum was determined by plating 10-fold serial dilutions onto BHI plates. Mice were sacrificed 4, 8 and 24 h post-inoculation and blood was collected aseptically via incision of the abdominal vena cava. Naïve mice were housed in the same facility and sacrificed for blood collection at the same time points as infected mice. Sera were isolated from blood using BD Microtainer SST tubes (Becton Dickinson). All sera (infected and naive mice) were filter-sterilized using PVDF 0.22 μm centrifugal filter units (MilliporeSigma) and were stored at −80°C.

### Lipid extraction

Lipid Extractions were performed using the MTBE protocol (36). Ten microliters of serum sample were added to 75 μl of LC-MS grade water, 5 μl Splash Lipidomix internal standard mixture (Avanti Polar Lipids; PN 370707), 575 μl MTBE, and 160 μl MeOH. Samples were vortexed at room temperature for 30 min, 200 μl water was added to break the monophase, followed by centrifugation at 17,000 *g* at RT for 3 min. The upper layer was transferred to a new Eppendorf vial. The extraction was then repeated with 300 μl MTBE, 100 μl MeOH, and 100 μl water, shaking at RT for 15 min, and centrifugation at 17,000 *g* for 3 min. The upper layers were combined, evaporated under vacuum, and reconstituted in 100 μl of mobile phase A for LC-FAIMS-MS/MS analysis.

### LC-FAIMS-MS

Normal-Phase/HILIC chromatographic separation was performed using a Dionex 3000 capillary HPLC comprising a binary pressure gradient pump and degasser, and an external column oven. Separation was performed using a Waters Acquity BEH Amide 1 × 150 mm column (Waters Corp; P/N 186004850) operated at 45°C. Mobile phase A consists of 80:20 hexane: IPA with 0.2% formic acid and 0.028% ammonia, and mobile phase B consists of 80:20 IPA: water with 0.2% formic acid and 0.028% ammonia. The flow rate was 25 μl/min, with 10 μl/min delivered to the mass spectrometer via a post-column split. The gradient is as follows: 0–1 min 0% B, 1.1–15 min 10%–30% B, 15–35 min 30% B, 35.1–45 min 85% B, 45.1–60 min 0% B.

Mass Spectrometry analysis in data-dependent acquisition mode (DDA) was performed using a Thermo Scientific Orbitrap Exploris 240 mass spectrometer (Thermo Scientific) equipped with a Thermo Scientific FAIMS Pro Duo interface. The instrument was operated in the negative-ion mode. Typically, electrospray ionization was carried out with a spray voltage of −3200 V and the FAIMS carrier gas flow was set at 0.7 L/min. Three FAIMS compensation voltages (CV) of 29 V, 34 V, and 39 V were used to allow comprehensive analysis of all phospholipid species. CVs were determined by infusing Splash LipidoMix into the instrument source at a flow rate of 5 μl/min and scanning from −100 V to 100 V. Unscheduled parallel reaction monitoring (PRM) mass spectrometry was carried out unscheduled with 2 microscans, an RF lens setting of 110, and a high energy collisional dissociation (HCD) setting of 35%.

### Data processing and statistical analysis

Untargeted and targeted lipidomics approaches were performed to profile the major lipids from pooled serum samples. Raw data were either processed using FreeStyle (Thermo) to extract MS/MS spectra for structural characterization or Skyline v.22.2.0.351 to generate the normalized peak areas of pre-selected lipid species for quantitative analysis, in which each subclass of lipids was normalized using Splash LipidoMix as their corresponding surrogate internal standards (37).

For lipid identification, we developed an MS/MS spectrum matching algorithm against an in-house library (supplemental Table S1). For quantitative analysis, we constructed a Skyline transition list based on the identified lipids and selected 15 SM species (supplemental Tables S2 and S3). The peak areas were normalized against with the spiked standards in the same subclass. For example, all PC species and ether PC species were normalized using the peak area of PC (15:0-18:1)-d7. The normalized peak areas were exported for further data analysis. We used GraphPad Prism (v10.1.2) for statistic analysis and data illustration. The generated Volcano plot, using a fold change (FC) threshold of 2.0 and adjusted *P* value threshold of 0.05.

## Results

### Optimal balanced CV values of reference standards

Because FAIMS acts as an ion filter by transmitting selected ions, we need to obtain CV spectra to determine the CVs that are suitable for transmitting all phospholipids. In this study, a CV scan experiment was performed on Splash LipidoMix lipid standards, which was directly infused into an Exploris 240 mass spectrometer that was coupled to an FAIMS device. The obtained CV spectra, by scanning the compensation voltage from 0 to 50 V over the range of *m/z* 400–1400, are shown in supplemental Fig. S1. The data indicated that the appropriate CVs for LPE, LPC, PA, PE, PG, PS, SM, PC, and PI were 39 V, 34 V, 39 V, 34 V, 34 V, 34 V, 29 V, 29 V, and 34 V, respectively. As highlighted in supplemental Fig. S1, the phospholipids of interest can be detected by operating FAIMS at 3 discrete values of CV, i.e., 29 V, 34 V and 39 V, which are referred to as optimal balanced CVs in this study, rather than scanning the CV across a wide range. This is extremely important when coupling LC-MS with FAIMS because the peak widths of LC are typically less than 12 s and it is important to obtain as many MS/MS spectra as possible for lipid identification. The CV spectra reveal that the ion mobilities of PC and SM are similar and significantly different from that of PA, PE, PG, PS, and PI, which most likely resulted from the impact of head groups since both PC and SM have a choline head group attached to a phosphate. We also observed a 5 V-increment of the optimal CV between diacyl-phospholipids and their corresponding lyso-forms.

### Reproducibility in reference standard analysis

Because the optimal balanced CVs of lipids can be affected by the composition of the matrix, we carried out NPLC-FAIMS-MS experiment with Step-CV scan, i.e., 23 V, 25 V, 27 V, 29 V, 31 V, 33 V, 35 V, 37 V, 39 V, 41 V, and 43 V. The obtained CV spectra, peak areas versus CVs are shown in supplemental Fig. S2, in which the peak areas at different CV values were integrated using Skyline. The result confirmed that the selection of 3 discrete CVs was suitable for NPLC-MS as well. With the selection of 29 V, 34 V and 39 V as the CV values, the reproducibility of the proposed NPLC-MS for quantitation is evaluated with 5 injections. Without any normalization, the relative standard deviation (RSD) of peak area for each lipid standard ranged from 14% to 26% (supplemental Fig. S2), and the RSD of retention time for each lipid was between 0.69% and 2.1% (supplemental Fig. S3).

### Optimal balanced CVs for serum lipid analysis

**Fig. 1:**
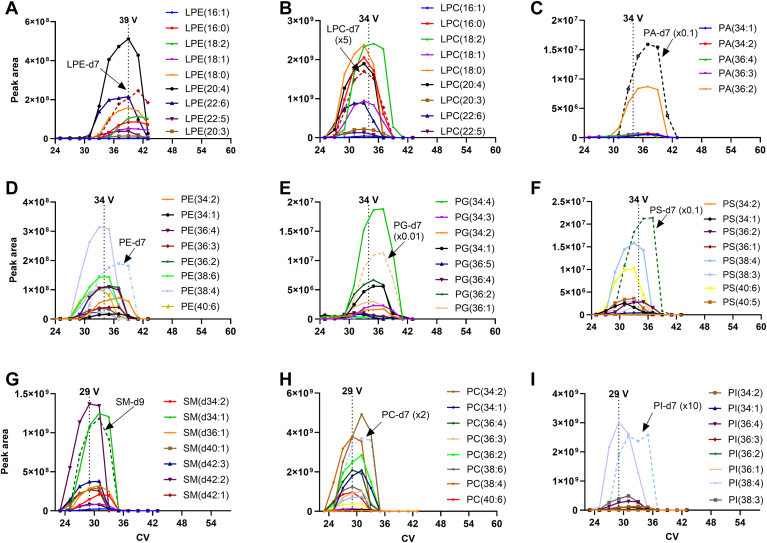
CV spectra from NPLC-FAIMS-MS analysis of pooled serum samples spiked with Splash LipidoMix. A: Peak areas of different compositions of LPEs; (B) Peak areas of different compositions of LPCs; (C) Peak areas of different compositions of PAs; (D) Peak areas of different compositions of PEs; (E) Peak areas of different compositions of PGs; (F) Peak areas of different compositions of PSs; (G) Peak areas of different compositions of SMs; (H) Peak areas of different compositions of PCs; (I) Peak areas of different compositions of PIs. The data were generated using Skyline.

It is well known that the mobility of an ion in a carrier buffer gas is related to its size and shape. For phospholipids, in addition to the difference among their head groups, minor structural differences among individual lipid species, such as the number, position, and geometry of double bonds in acyl chains, can have an impact on their mobility. In other words, the optimal CV setting that selectively transmits standard lipids through the interface into the mass spectrometer may not be suitable for those serum lipids that are structurally different from reference lipids. To this end, we performed NPLC-MS under step CV scan setting for pooled serum samples. The obtained CV spectra, i.e., peak areas versus CVs, were illustrated in Fig. 1. The results confirmed that the optimal balanced CV values for standard lipids were also suitable for the corresponding classes of lipids from serum except PI. The optimal CV for PI (15:0-18:1)-d7 is 34 V; however, the optimal balanced CV for those PIs from serum is 29 V (Fig. 1I). Overall, the optimal CVs of lipids from mouse sera shifted toward a smaller value for diacyl-phospholipids, comparing to their corresponding standard. The results indicate a correlation between optimal CVs and acyl chains, i.e., (1) the longer the acyl chain the smaller optimal CVs; (2) the greater number of double bonds the larger the optimal CV. The detected lipids from sera samples typically have longer acyl chains and more double bonds than that of reference standard, total number of carbon atoms of 33 and 1 double bond. Interestingly, no significant shift was observed in the optimal CVs for ether-linked phospholipids (supplemental Fig. S4).

### Profile of lipids in mouse serum

**Fig. 2:**
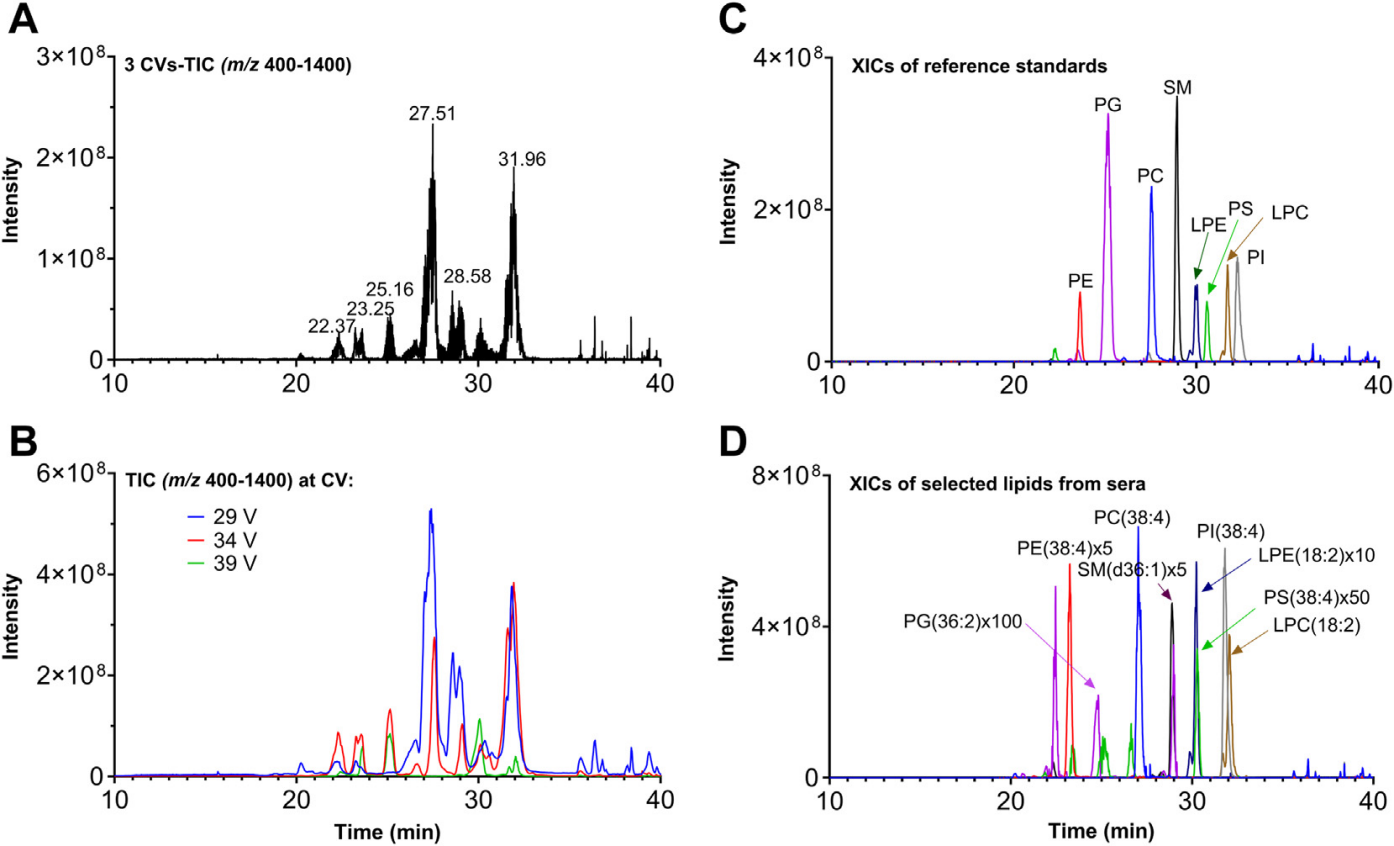
NPLC-FAIMS-MS analysis of pooled sera spiked with Splash LipidoMix. A: Total ion chromatogram (TIC, *m/z* 400–1400); (B) TIC (*m/z* 400–1400) at CV = 29 V, CV = 34, and CV = 39 V; (C) Extracted ion chromatograms of 8 standards in Splash LipidoMix; (D) Extracted ion chromatograms of 8 serum lipids.

We then validated the method by analyzing pooled mouse sera from 3 control animals and 5 infected animals (24 h after inoculation) at 3 CVs. The obtained results were presented in Fig. 2, in which Fig. 2A shows the total ion chromatogram (TIC) from all 3 CV values, together with the TICs at CV = 29 V, CV = 34 V, and CV = 39 V (Fig. 2B). The extracted-ion chromatograms of 8 standards and 8 serum lipids are illustrated in Fig. 2C, D, respectively. The extracted MS spectra for each subclass of phospholipids are presented in supplemental Fig. S5. The data show that the HILIC under normal phase chromatography conditions can separate lipids primarily based on lipid classes, and the elution patterns are based largely on the properties of the phospholipid head group, although the total number of carbon atoms and double bonds also has a minor effect on retention time.

Although the purpose of using FAIMS device is not to separate individual lipid species, the FAIMS can provide a certain degree of additional dimension separation for the same subclass of lipids based on the combination of acyl length and number of double bonds as demonstrated in supplemental Figs. S6 and S7. For example, LPC (18:0) is the major species that was detected at CV = 27 V (supplemental Fig. S6A); whereas it almost disappeared when CV was changed to 37 V and LPC (18:2) is the predominant species (supplemental Fig. S6F). On the other hand, we can use 34 V as the optimal balanced CV to permit most of LPC species to pass through FAIMS device. For diacyl-PCs, 34 V is the most favorable CV for PC (34:2), only least favorable for PC (38:4) (supplemental Fig. S7). Similarly, an optimal balanced CV value of 29 V can be used for the determination of most PC species. These results demonstrate that the coupling FAIMS device to NPLC-MS provides a powerful means to prevent mass spectrometers from contamination and increase productivity.

### Reproducibility in analyzing serum lipids

We evaluated the reproducibility of the proposed method for the quantification of the abundance of lipids in pooled serum samples. The LipidoMix reference standards were spiked into samples prior to lipid extraction. This normalization is to make samples more comparable and measure differences in the abundance of lipids that are associated with *A. baumannii* infection rather than bias or errors. The reproducibility of normalized peak areas of representative lipids for 3 different days, Day 1, Day 2, and Day 3, was illustrated in supplemental Fig. S8. Data was from 3 independent NPLC-FAIMS-MS/MS runs for each day. The results indicate that the RSD for each day ranges from 1.2% to 20%. We also calculated the day-to-day reproducibility with the average of normalized peak areas of 3 different days and the RSD ranges from 4.7% to 9.1% (supplemental Fig. S9). Thus, we demonstrate that the NPLC-FAIMS-MS/MS method can be used for the quantitative assessment of changes in the abundance of lipids in complex serum samples.

### NPLC-FAIMS-MS/MS for lipid identification

**Fig. 3:**
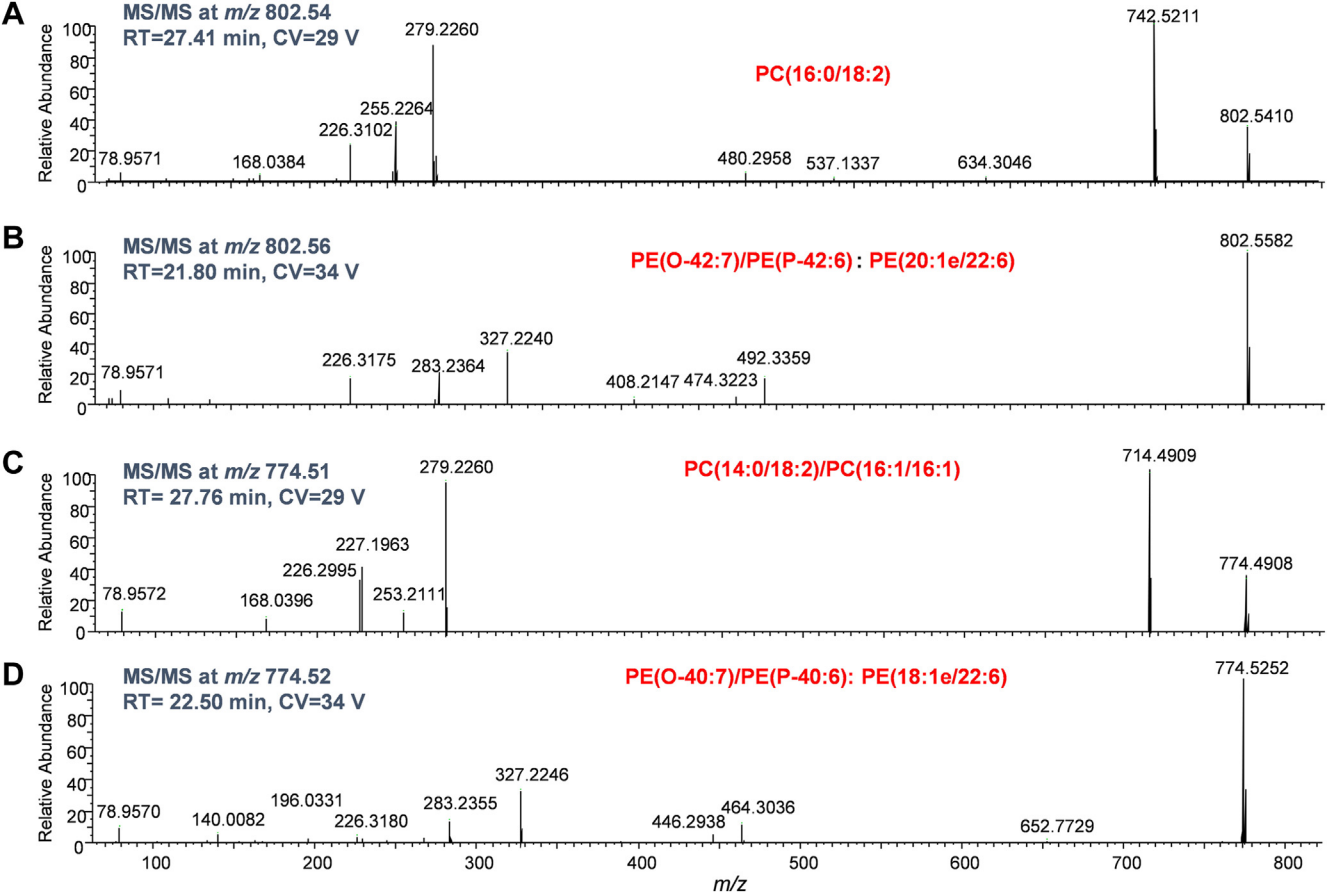
Extracted MS/MS spectra generated by DDA from NPLC-FAIMS-MS/MS. A: MS/MS spectrum of precursor at *m/z* 802.54, CV = 29 V; (B) MS/MS spectrum of precursor at *m/z* 802.56, CV = 34 V; (C) MS/MS spectrum of precursor at *m/z* 774.51, CV = 34 V; (D) MS/MS spectrum of precursor at *m/z* 774.53, CV = 34 V.

Phospholipid molecular species are often expressed as isobaric species that are denoted by the phospholipid class, the total number of carbon atoms, and double bonds contained in the esterified fatty acyl groups (e.g., PC (34:2) as sum composition or shorter name annotation) (38). PC 34:2 can consist of several isomeric compositions, e.g., PC (16:1-18:1) and PC (16:0-18:2). In addition to the fact that lipids may have exactly the same mass (isomers), they can also have nearly the same mass (isobars). For example, the deprotonated formate adduct of PC (34:2) produces ions at *m/z* 802.5604; while the deprotonated PE (20:1e/22:6) (alkenyl ether phosphatidylethanolamine) produces ions at *m/z* 802.5753. In MS1 mode, we can take advantage of high-resolution accurate mass offered by Orbitrap for quantitation. However, isobars may become a significant issue for MS/MS experiments because the resolution of precursor isolation is typically achieved by a low-resolution quadrupole, with a typical 0.4–5 Th window in the Orbitrap. In this study, an isolation window of 2 Th for precursors in MS/MS experiment was used. As illustrated in Fig. 3, NPLC-FAIMS-MS/MS experiments overcome the complexity of the isobaric interference by offering two additional-dimensional separations, i.e., LC and FAIMS. Figure 3A, B present the extracted MS/MS spectra for the precursor ions at *m/z* 802.54 and *m/z* 802.56, respectively. These MS/MS experiments were acquired in DDA mode, and the corresponding compensation voltages were at 29 V and 34 V at 27.41 and 21.80 min, respectively. The diagnostic fragment ions for the PC head group are *m/z* 742.5611 and *m/z* 168.0384 (Fig. 3A), together with fragment ions associated with two acyl chains, i.e., *m/z* 255.2264 for C16:0 and *m/z* 279.2260 for C18:2. On the other hand, only the fragment ions associated with the neutral loss of *sn*-2 acyl chain and the *sn*-2 residue itself (*m/z* 492.3359 and *m/z* 327.2240) were observed for ether PE. It is worth mentioning that the fragment ions at *m/z* 283.2364 arise from *m/z* 327.2240 by consequential loss of CO_2_, rather than anion ions corresponding to C18:0 fatty acid (*m/z* 283.2646). Figure 3C, D show the MS/MS spectra of precursor ions at *m/z* 774.51 and *m/z* 774.52 extracted at RT = 27.76 min, CV = 29 V, and RT = 22.50, CV = 34 V, respectively. The mass spectrum in Fig. 3C shows a fragmentation pattern, i.e., *m/z* 714.4909 and *m/z* 168.0396 as the characteristic fragments of PC, whereas *m/z* 227.1963, *m/z* 253.2111 and *m/z* 279.2260 for the evidence of C14:0, C16:1, and C18:2 fatty acids, which allows unequivocal identification of the presence of two isomers, PC (14:0-18:2) and PC (16:1-16:1). The MS/MS spectrum in Fig. 3D confirmed the composition of this ether PE species is PE (18:1e-22:6).

We performed the NPLC-FAIMS-DDA to examine the lipidome of mouse serum after *A. baumannii* infection, including 4 h, 8 h, 24 h post-inoculation (5 each) and their corresponding naïve controls (n = 3 each). In order to identify the detected lipids and construct Skyline transition list for quantification, we built an in-house tool, LipiDect. Briefly, LipiDect software generated a library for >2,600 unique lipids that is adapted from LipidBlast (39), https://systemsomicslab.github.io/compms/index.html, which contains the theoretical *m/z* of the precursors, fragment ions, expected retention time ranges and CVs (supplemental Table S1). For identification, each observed precursor ion (MS1 spectrum) and its fragment ions (MS/MS spectra) are matched to the *m/z* ions in the library. The criteria for matching include high-mass accuracy (10 ppm) for both precursor and fragment *m/z* tolerance; being within the expected RT range; having the expected CV. The highest matching spectrum for each lipid moiety (based on the percentage of the observed peaks of the spectrum that matched the theoretical peaks) is considered the primary match and all remaining matching spectra are considered secondary matches.

The “short name” annotation in LipidBlast or “Sum compositions” in other Lipidomics database specifies the information on the number of carbons and the number of double bonds (10, 40). The characteristic fragment ions related to fatty acids were used to identify the acyl chain compositions, termed as “name” annotation in LipidBlast, “Fatty Acid Identification” in LiLA (40), or “Fatty Acyl Identification” in LipidNovelist (10). It is important to point out that the MS/MS data from our DDA experiments were not efficient in annotating double bond positions, stereospecificity and regiospecificity. Nevertheless, we were able to annotate a total of 266 lipids from mouse sera, including 24 h controls and 24 h post-inoculation, with the information on the composition of acyl chain lengths and double-bond counts (supplementary Table S2). Unfortunately, we were not able to identify the acyl chain compositions for sphingomyelins due to lack of diagnostic fragment ions from MS/MS experiments. Additional information from MS^3^, MS^4^, *etc.*, spectra are needed for identifying SM. For example, MS^3^ spectra were used for determining the long chain base and fatty acyl chain moieties from the formate adducts of SM (41).

### Temporal profile of serum lipids after *A. baumannii* inoculation

It is well known that bacterial infection can alter serum lipids (28, 29). LPC has been proposed as a preventative therapy for patients at risk of severe infections caused by *A*. *baumannii* (42, 43, 44). To further evaluate our NPLC-FAIMS-MS/MS method, we investigated the temporal profile of serum lipids after *A. baumannii* inoculation.

After verifying the identification, a transition list was produced that is ready to be used as a Skyline transition list to perform quantitative data analysis, using only MS1 for quantitation. Because the MS/MS spectra of SMs did not provide the required information to identify their acyl compositions, the initial Skyline transition list for SMs was adapted from LipidBlast. From the analysis results, 15 SM species were kept in the final Skyline transition list (supplemental Table S3). All 15 SMs have a signal-to-noise (S/N) ratio of peak intensity greater than 3. We processed the NPLC-FAIMS-DDA data for the sera from the mice with *A. baumannii* at 4 h, 8 h and 24 h post-inoculation and their corresponding naïve controls. The peak boundaries and CVs for integrating targeted molecules are shown in supplemental Tables S3, S4-1, S4-2, and S4-3 show the results for sera 4 h, 8 h, and 24 h after inoculation, respectively. These tables contain raw data, validated data, re-formatted data, and *t* test results. The validation criteria include RT window and average mass error. We excluded the data from the tables if its mass error is greater than 5 ppm.

**Fig. 4:**
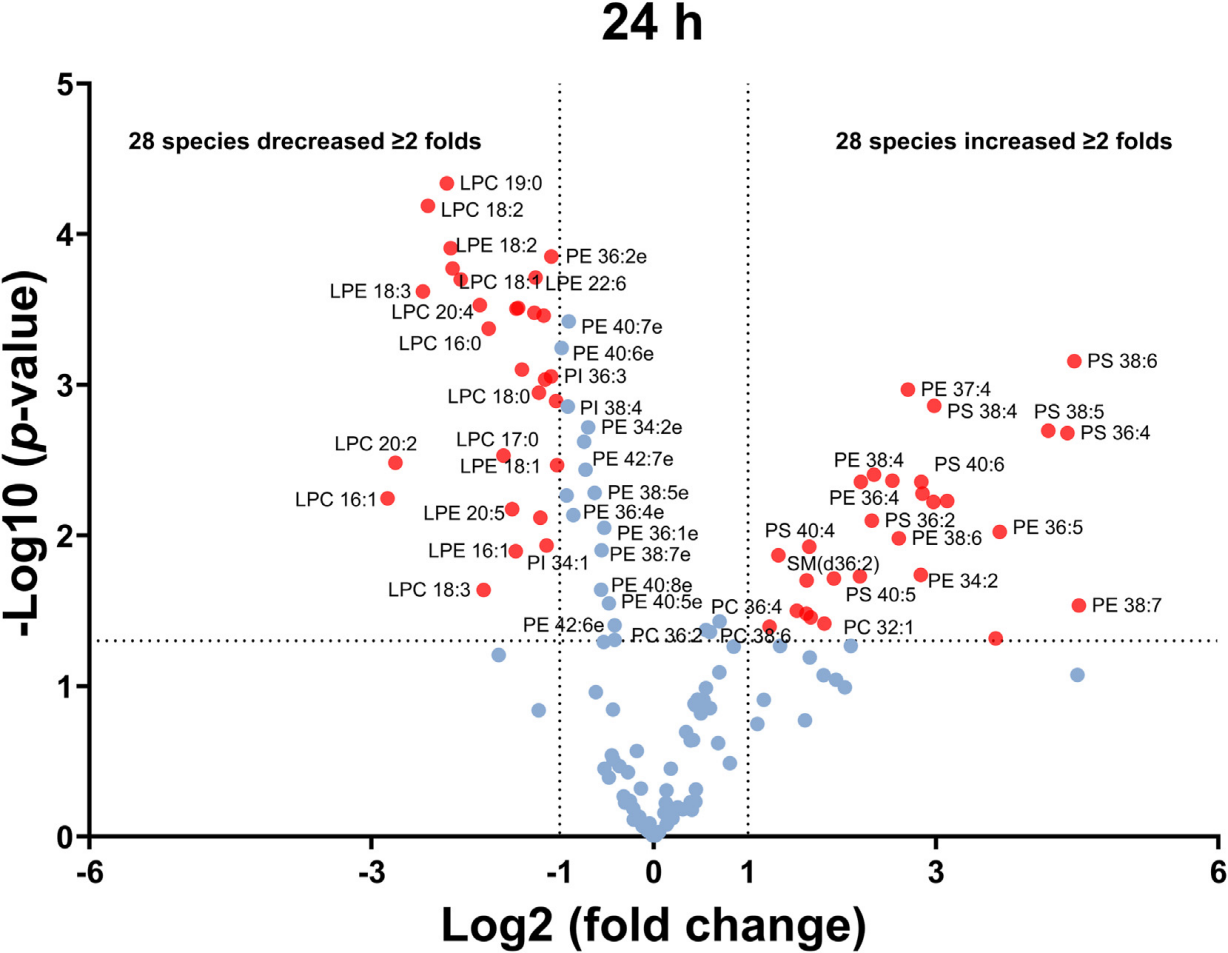
Volcano plots demonstrating the effect of *A. baumannii* infection on the circulating lipidome from the mouse serum. Data were from 3 controls and 5 infected animals, 24 h after inoculation. The red spots highlight those with a fold change ≥ 2 and adjusted *P*-value ≤ 0.05. Statistical analysis was carried out using Multiple unpaired t tests (GraphPad Prism v10.2.3).

We used GraphPad Prism for statistic analysis and data illustration, and the Volcano plots representing the lipidome changes at 4 h and 8 h post-inoculation are illustrated in supplemental Fig. S10A, B, respectively. The volcano plot for 4 h post-inoculation shows the altered lipid species consisting primarily of PI, PC, and PS. Surprisingly, it was revealed that PI 38:4 (18:0-20:4) was decreased; whereas PI 34:1 (16:0-18:1) was increased in the sera 4 h after inoculation (supplemental Fig. S10A). We speculate that this observation may be associated with the initiation of the host inflammation response to the infection. All PIs are decreased 8 h after inoculation, in addition to the alterations in levels of ether PC and ether PE species (supplemental Fig. S10B). The lipidome changes in 24 h post-inoculation are shown in Fig. 4. The resultant volcano plot indicates the level of 56 lipids was significantly changed, including 28 increased and 28 decreased lipid species. While almost all decreased lipid species were LPCs and LPEs, most of the increased species contain PUFA, e.g., PE 37:4, PE 38:4, PE 38:6, PS 38:6, PS 38:4, and PS 36:4. MS/MS experiments indicated that their PUFAs were predominantly arachidonic acid (AA, 20:4) and docosahexaenoic acid (DHA, 22:6). This observation suggests that altered lipid metabolism in immune cells may be associated with various important inflammatory conditions.

**Fig. 5:**
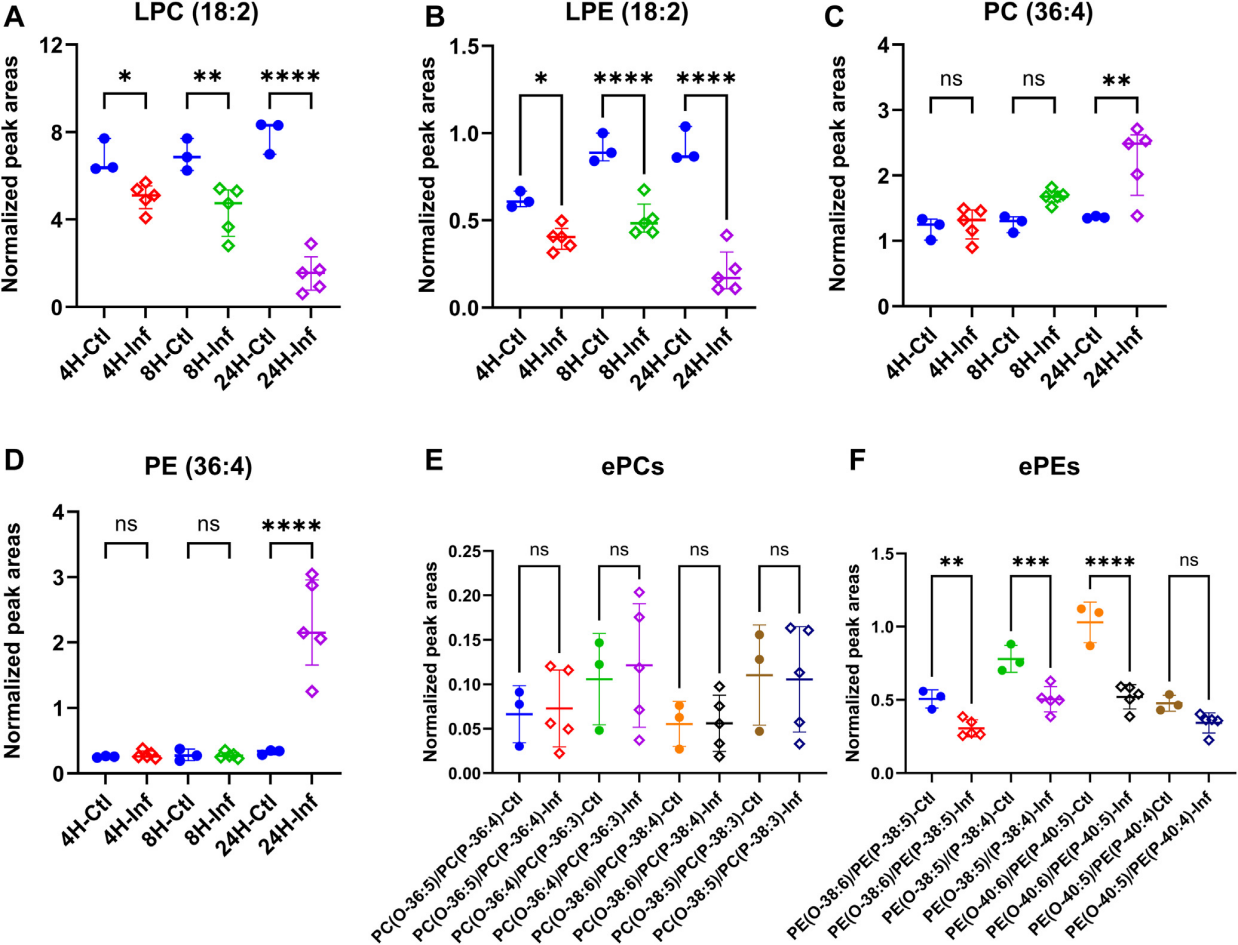
The effect of *A. baumannii* infection on the profiles of four major serum phospholipids. Comparison of the relative quantity of (A) LPC (18:2), (B) LPE (18:2), (C) PC (36:4), and (D) PE (36:4) from controls to infection at 4 h, 8 h, and 24 h. Comparison of the relative quantity of (E) ePCs and (F) ePEs between controls and 24 h after inoculation. The peak areas were normalized against 240 nmol of spiked LPC (18:1) (d7), 55 nmol of spiked LPE (18:1) (d7), 1065 nmol of spiked PC (15:0-18:1) (d7) and 55 nmol of spiked PE (15:0-18:1) (d7) in 10 μl mouse serum for LPC, LPE, PC and PE, respectively. For ether PCs, 1065 nmol of spiked PC (15:0-18:1) (d7) was used for peak area normalization. For ether PEs, 55 nmol of spiked PE (15:0-18:1) (d7) was used for peak area normalization. Statistical analysis was carried out using a One-way ANOVA (ns = not significant, ∗*P* = < 0.05, ∗∗*P* < 0.01, ∗∗∗*P* = < 0.001, and ∗∗∗∗*P* = < 0.0001).

The Volcano plots reveal significant changes of LPCs and LPEs in the mouse sera 24 h post-inoculation. To further assess these alterations, one-way analysis of variance (ANOVA) was used to compare the difference between 4 h, 8 h, and 24 h post-inoculation and their corresponding controls (Fig. 5). The results indicated that LPC (18:2) and LPE (18:2) levels were decreased as early as 4 h post-inoculation samples (Fig. 5A, B). The profiles of detected LPCs and LPEs at 4 h, 8 h, and 24 h are shown in supplemental Figs. S11 and S12, respectively. Notably, 11 LPC and 8 LPE species were significantly decreased in the sera 24 h after inoculation. In contrast, the levels of PC (36:4) and PE (36:4) were significantly increased in 24 h post-inoculation samples, although no significant changes were observed in 4 h and 8 h post-inoculation samples (Fig. 5C, D, respectively). The lipid profiles of PIs, PSs, PCs, and PEs were presented in supplemental Fig. S13.

### Analysis of ether lipids

The comparison of the relative quantity of ePCs and ePEs between controls and 24 h post-infection sera are presented in Fig. 5E, F, respectively. The results indicated that the ePC levels showed large variations among individual animals, and no significant changes were observed after inoculation. However, the ePE levels were relatively close in each group and significantly decreased for PE(O-38:6)/PE(P-38:5), *m/z* = 748.5287; PE(O-38:5)/PE(P-38:4), *m/z* = 750.5443; PE(O-40:6)/PE(P-40:5), *m/z* = 776.5600. It is worth pointing out that the ePE levels include all isomers and it is not possible to quantify individual isomers because parallel reaction monitoring (PRM) experiments would have to be performed.

**Fig. 6:**
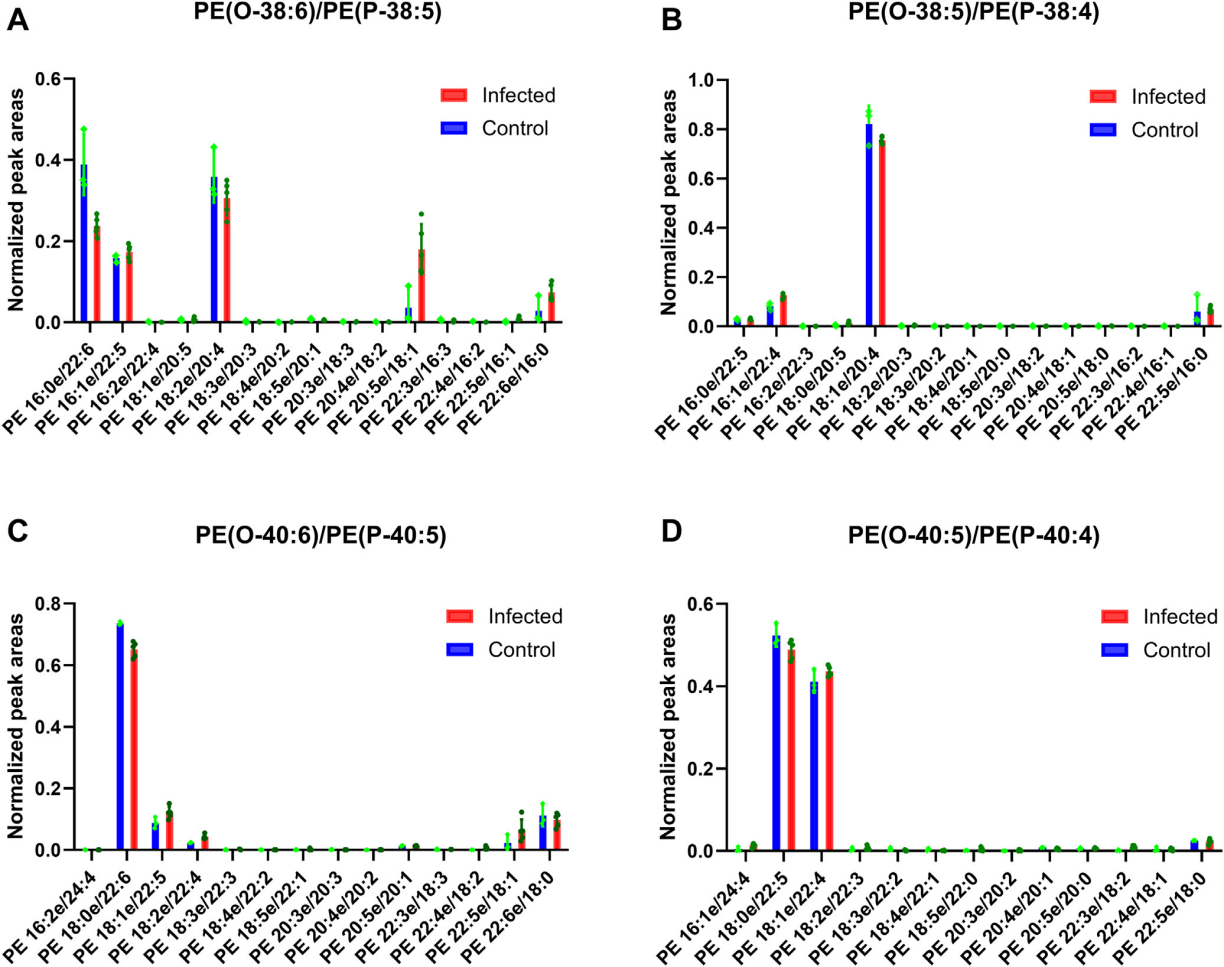
NPLC-FAIMS-PRM analysis for identifying isomeric ether PEs. (A) normalized peak areas of different compositions of PE(O-38:6)/PE(P-38:5), PRM precursor at *m/z* = 748.5287; (B) Normalized peak areas of different compositions of PE(O-38:5)/PE(P-38:4), PRM precursor at *m/z* = 750.5443; (C) Normalized peak areas of different compositions of PE(O-40:6)/PE(P-40:5), PRM precursor at *m/z* = 776.5600; (D) Normalized peak areas of different compositions of PE(O-40:5)/PE(P-40:4), PRM precursor at *m/z* = 778.5756. Peak areas were normalized against the total peak areas of all detected isomeric compositions produced from the ePE isomers.

In order to profile the changes of isomeric ePE and ePC species, we carried out targeted lipidomics analysis, i.e., NPLC-FAIMS-PRM experiments. Fig. 6A–D show the relative quantity of each isomer of PE(O-38:6)/PE(P-38:5), PE(O-38:5)/PE(P-38:4), PE(O-40:6)/PE(P-40:5) and PE(O-40:5)/PE(P-40:4), respectively. The results suggest that PE(O-38:5)/PE(P-38:4) is dominated by one isomeric species, i.e., PE 18:1e/20:4; wheareas PE(O-38:6)/PE(P-38:5) consists of 5 isomers and PE(O-40:5)/PE(P-40:4) has 2 major isomers. To further assess the alterations in these individual isomers, one-way ANOVA was performed. The results are presented in supplemental Fig. S14, in which the peak area of each isomer was normalized against the total area of all isomers of the same composition (ns = not significant, ∗*P* = <0.05, ∗∗*P* < 0.01, ∗∗∗*P* = <0.001, and ∗∗∗∗*P* = <0.0001).

## Discussion

Orbitrap mass spectrometers offer high levels of accuracy, scanning speed, and sensitivity, and have been widely used in proteomics research. Although their applications towards lipidomics have grown tremendously, it is still challenging due to the extreme complexity of lipidomics samples. It is reasonable to believe that this is due to the contamination when switching from lipid analysis to peptides or proteins, and then back again. FAIMS interface can improve workflow robustness because it blocks neutrals and unwanted ions in the source to the entrance of the mass spectrometer. Furthermore, the FAIMS device can be added on or taken off without breaking the instrument vacuum, which makes it easy to clean and install. Therefore, it is highly desired to couple a FAIMS device between liquid chromatography equipment and a mass spectrometer for analyzing complex samples. For FAIMS-based applications, the selection of compensation voltages is key to success. In this study, we first obtained CV spectra by direct infusion of Splash LipidoMix standards, from which 3 CVs were chosen for further evaluation. Using the pre-selected CVs, i.e., 29 V, 34 V, and 39 V, we performed NPLC-FAIMS-MS on pooled serum samples, spiking with lipid standards.

While RPLC can separate lipids with different fatty acyl chains, HILIC provides separation based on headgroup polarities. The mobile phases A and B in a typical HILIC system consist of formic acid or NH_4_HCO_3_ in water and ACN (10). However, our unique NPLC system consists of a HILIC column with a normal phase gradient that also contains water. Mobile phases A and B consist of 80:20 hexane:IPA and 80:20 IPA:water, respectively, with formate and ammonium ion pairs. This mobile phase system separates phospholipids by headgroups and uses water, like HILIC, but the unique composition of the mobile phase with IPA in both A and B allows the mixing of hexane with water, while at the same time avoiding the use of typical normal phase solvents like dichloromethane or acetone. It is friendly to ESI, and also allows this method to be used for lipids that have poor solubility in acetonitrile, such as endotoxin (lipid A).

For annotating serum lipids, we developed a database-matching algorithm, LipiDect. This software can search MS/MS data using a customized database for acyl chain identification from fragment ions. The sum compositions were then derived from accurate parent ions, and used to generate a transition list for Skyline to perform semi-quantitative analysis of selected lipid species. We also evaluated NPLC-FAIMS-PRM for a more accurate analysis of isomeric ether lipids, i.e., ePE and ePC.

We then applied the proposed method to investigate the effect of *A. baumannii* infection on mouse serum lipidome. We compared the mouse serum collected from different times post-inoculation, i.e., 4 h, 8 h, and 24 h, with their corresponding controls. We found an early and sustained decrease in PI 38:4 (18:0-20:4) species in the sera of infected mice as early as 4 h; whereas PI 34:1 (16:0-18:1), several PC and PS species were increased. This discrepancy may result from the fact that phosphoinositides are an important class of signaling lipids and they are involved in host inflammatory and immune responses. We speculate that the observation may be associated with the initiation of the host inflammation response to the infection, e.g., increased consumption of PUFAs. It has been reported that cytosolic phospholipase A2 (cPLA2) of macrophages can be activated following exposure to lipopolysaccharide, and has exquisite specificity toward AA 20:4 from PI (45). Especially, AA 20:4 is the major PUFA in the membrane of innate immune cells and is not uniformly distributed among membrane glycerophospholipids. At 8 h after inoculation, the levels of 22 lipids in mouse sera were significantly decreased, including PI, PE, PC, ePE, and ePC species. This phenomenon may be explained by the fact that *A. baumannii* infection induces clinical symptoms within 8 h of infection, and lung tissue damage thereafter (34).

The results from 24 h post-inoculation animals indicate 28 species were increased and 28 species were decreased in the sera of infected mice at this time point. The serum levels of LPC and LPE, especially LPC (18:2), were significantly lower than those controls. This observation is consistent with previous reports that LPC levels are lower in mouse sera after bacterial infection (28, 46). LPC is predominately produced endogenously through a set of different processes including from the cleavage of PC via the action of phospholipase A. LPC can be converted back to PC by the enzyme LPC acyltransferase. The changes in the content ratio of PC-LPC can be modulated by mammalian cells’ *de novo* cyclical synthesis and degradation of PC as part of the Lands cycle (47). Of the 28 increased species, 14 PE and 8 PS species contain PUFAs. When further investigating the acyl chain composition, it is surprising that 11 PE and 7 PS species contain either AA (20:4) or DHA (22:6). Our findings reveal dynamic changes in the serum lipidome during *A. baumannii* infection, suggesting that lipids play important roles in host inflammatory and immune responses. This is in agreement with previous reports that macrophages produce free PUFAs, which are the precursors of the proinflammatory eicosanoids and related bioactive lipids, while also mediating repair through an anti-inflammatory response (48). Furthermore, phospholipase A2 of macrophages is activated after 1 h KDO-Lipid A treatment (45). Thus, we speculate that the immune cell populations at 24 h after *A. baumannii* infection were completely different from those at 4 h (M1 macrophages), for example, a new phenotype of macrophages (M2 macrophages), neutrophils, or other cells involved in lung host defense. A study in an in vivo rat sepsis model showed increases in PE levels, including PE 16:0-22:6 and PE 18:0-22:6, in the sera 4–12 h after the induction of sepsis (49). *A. baumannii* can cause infections in the lungs (pneumonia). Thus, immune responses in the lung may attribute to the increase of PUFA-containing PE and PS. The mechanism for the generation of PUFA-containing phospholipids was believed to be through the Lands pathway, which incorporates PUFAs into individual molecular species of lysophospholipids through the action of lysophospholipid acyltransferases (47). Our data show a clear increase in PUFA-containing PE and a decrease of LPE. This association strongly suggested that PE was generated by the reacylation to LPE through the Lands cycle. Surprisingly, the level of PUFA-containing PC species only marginally increased (less than two folds), although the level of LPC decreased by greater than twofolds. One possible explanation for this phenomenon is the downregulation in LPC acyltransferase gene expression. On the other hand, the level of PUFA-containing PS species increased significantly. It is, therefore, possible that PUFA-containing PC species, generated from LPC through the remodeling of PC fatty acyl composition, might be further converted to PS species. Indeed, PS can be synthesized from PC and PE by two base-exchange enzymes, PS synthase-1 (PSS1) and PS synthase-2 (PSS2). PSS1 exchanges serine for choline of PC, whereas PSS2 exchanges ethanolamine of PE for serine (50). Unfortunately, no information is available on the transcriptional regulation of expression of the LPC acyltransferase gene and PS synthase gene. It is noteworthy that PS can be converted to PE by PS decarboxylase, a mechanism to control the levels of PS and PE. PS exposure on the cell surface is an early event in apoptosis, which is externalized during apoptosis and originates from a pool of newly synthesized PS (51). Previously, the cells involved in lung host defense, including alveolar macrophages, bronchial epithelial cells, and alveolar type II cells, have been isolated from human subjects, where lipidomic analysis by LC-MS/MS was performed (52). It was revealed that the human bronchial epithelium is highly enriched in diacyl AA (20:4)-containing phospholipids. Hence, the bronchial epithelium could be another source of detected PUFA-containing PE species, as a result of lung tissue damage caused by *A. baumannii* (34). However, the biological significance of our findings and biosynthetic pathways will require further investigation.

In summary, we have demonstrated that the proposed NPLC-FAIMS-MS/MS is a valuable technique for Orbitrap-based lipidomics. Although FAIMS is not able to completely separate different subclasses of phospholipids nor isomeric species, it affords a certain degree of separation that is highly valuable for structural confirmation. More importantly, it offers a means of protecting MS entrance from contamination, which enables an MS facility to share a platform to be used seamlessly for both proteomics and lipidomics. We envision that this workflow will further expand the application of Orbitrap instruments in lipidomics.

## Data availability

The mass spectrometry data have been deposited to the ProteomeXchange Consortium via the PRIDE partner repository with the dataset identifier PXD055224 (53). The data supporting this study are available from the corresponding author upon request.

## Supplemental data

This article contains supplemental data.

## Conflict of interest

The authors declare that they have no conflicts of interest with the contents of this article.

## Supplementary data

Table S4-3

LC-FAIMS-HRMS-SI-R2
