# Supplementary material for: Development of LC-FAIMS-MS and its application to lipidomics study of Acinetobacter baumannii infection
Source: J Lipid Res. 2024 Oct 10;65(11):100668. doi: 10.1016/j.jlr.2024.100668 (PMC11577210; doi:10.1016/j.jlr.2024.100668)
Supplement: LC-FAIMS-HRMS-SI-R2 [file mmc7.docx]

Development of NPLC-FAIMS-MS and its application to lipidomics study of *Acinetobacter baumannii* infection

Jianjun Li^1^, Jacek Stupak^1^, Arsalan S. Haqqani^1^, Greg Harris^1^, Hongyan Zhou^1^, Sam Williamson^1^, Rui Chen^1^, H. Howard Xu^2^, Wangxue Chen^1^

^1^Human Health Therapeutics Research Centre, National Research Council Canada, 100 Sussex Drive, Ottawa, Ontario, K1A 0R6, Canada. ^2^Department of Biological Sciences, California State University Los Angeles, Los Angeles, CA, USA.

**Table of Contents**

Fig. S1. Extracted CV spectra of Splash LipidoMix lipid standard.

Fig. S2. The means and standard deviations of the peak areas of Splash LipidoMix at 3 discrete CVs from NPLC-FAIMS-MS experiments (n = 5).

Fig. S3. Retention times of lipid standard in Splash LipidoMix.

Fig. S4. Peak areas of PC, ePC, PE and ePE at different CV values.

Fig. S5. Extracted MS spectra from NPLC-FAIMS-MS analysis of pooled serum samples.

Fig. S6. Extracted ion chromatograms of LPC (18:2), LPC (18:1) and LPC (18:0) at different CV values.

Fig. S7. Extracted ion chromatograms of PC (34:2), PC (36:4), PC (36:2), PC (38:6) and PC (38:4) at different CV values.

Fig. S8. Intraday reproducibility of normalized peak areas of representative lipids (n = 3).

Fig. S9. Day-to-day reproducibility of normalized peak areas of representative lipids from 3 different days (n = 3).

Fig. S10. Volcano plots demonstrating the effect of *A. baumannii* infection on the circulating lipidome from the mouse sera 4 h and 8 h after inoculation.

Fig. S11-1. Comparison of relative quantity of LPCs from controls to infection at 4 h, 8 h and 24 h, respectively.

Fig. S11-2. Comparison of relative quantity of LPCs from controls to infection at 4 h, 8 h and 24 h, respectively.

Fig. S12. Comparison of relative quantity of LPEs from controls to infection at 4 h, 8 h and 24 h, respectively.

Fig. S13. The impact of *A. baumannii* infection on serum lipid profiles, comparison of relative quantity of lipids between controls and 24 h after inoculation.

Fig. S14. NPLC-FAIMS-PRM experiment for investigating the effect of *A. baumannii* infection on isomeric PEs, comparison between controls and 24 h after inoculation, respectively.

Table S1. Library for LipiDect. This table contains the theoretical *m/z* of the precursor and fragment ions and containing expected retention time ranges and CVs.

Table S2. Lipid ID. This table contains the identified lipids with the information on subclasses, total number of carbons and total number of double bonds, the fatty acyl identification.

Table S3. Transition List and Peak Boundaries. This file contains the transition list that can be inserted in Skyline for quantitative analysis. It also contains Peak Boundaries and CVs that can be imported to Skyline for peak integration to process the data from 4 h, 8 h and 24 h sera after inoculation.

Table S4-1. Skyline report and statistic analysis result in sera 4 h after inoculation. The file contains the information on Total Area. Normalized Area, Average Measured Retention Time, Library Ion Mobility Value, Normalization Method, Average Mass Error PPM and RAW File Name.

Table S4-2. Skyline report and statistic analysis result in sera 8 h after inoculation. The file contains the information on Total Area. Normalized Area, Average Measured Retention Time, Library Ion Mobility Value, Normalization Method, Average Mass Error PPM and RAW File Name.

Table S4-3. Skyline report and statistic analysis result in sera 24 h after inoculation. The file contains the information on Total Area, Normalized Area, Average Measured Retention Time, Library Ion Mobility Value, Normalization Method, Average Mass Error PPM and RAW File Name.


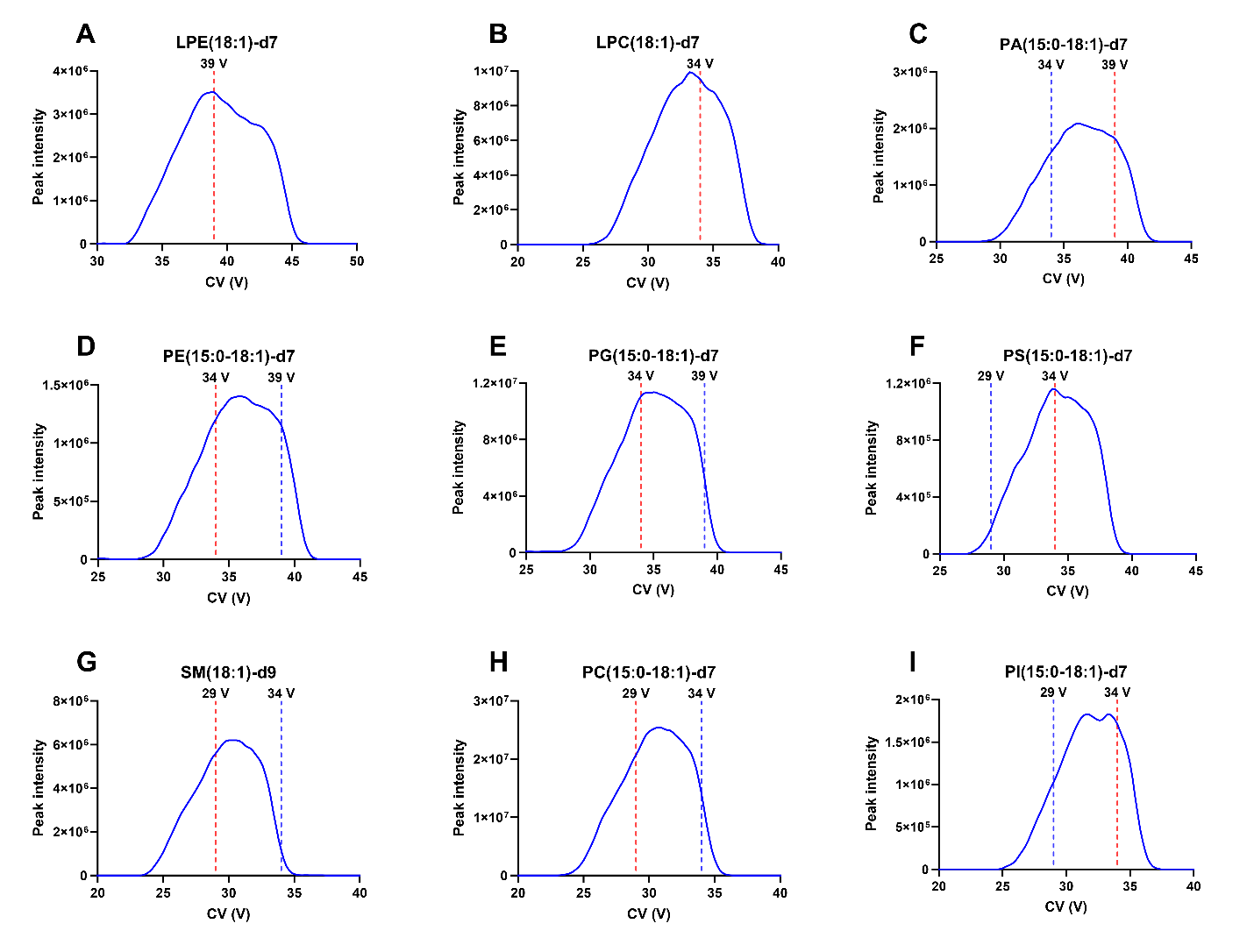


Fig. S1. Extracted CV spectra of Splash LipidoMix lipid standard. (A) *m/z* 485.3379, LPE (18:1)-d7; (B) *m/z* 573.3903, LPC (18:1)-d7; (C) *m/z* 666.5097, PA (15:0-18 :1)-d7; (D) *m/z* 709.5519, PE (15:0-18 :1)-d7; (E) *m/z* 740.5464, PG (15:0-18 :1)-d7; (F) *m/z* 753.5417, PS (15:0-18 :1)-d7; (G) *m/z* 782.6379, SM (18:1)-d9; (H) *m/z* 797.6043, PC (15:0-18:1)-d7; (I) *m/z* 828.5625, PI (15:0-18:1)-d7.


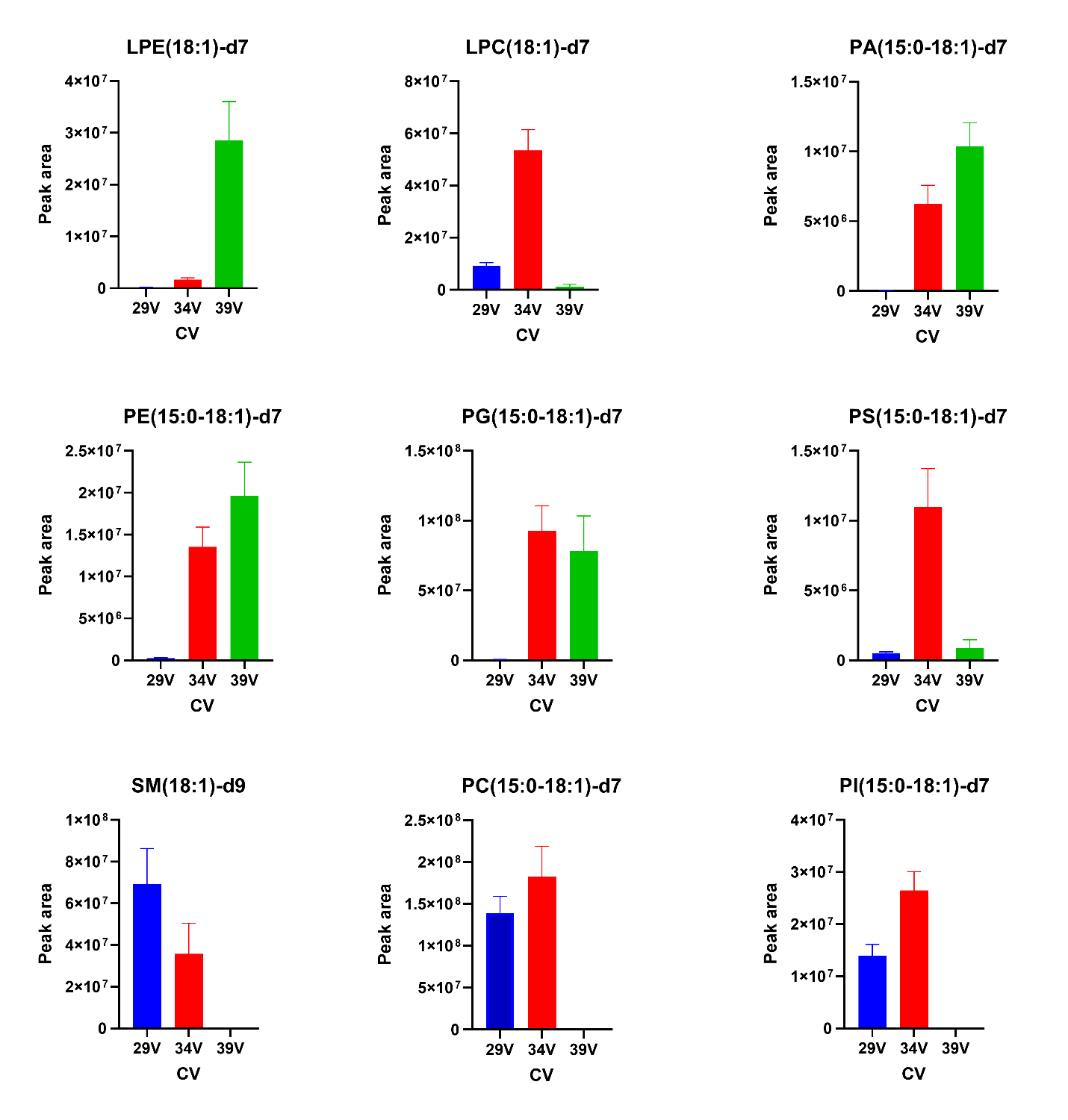


Fig. S2. The means and standard deviations of the peak areas of Splash LipidoMix at 3 discrete CVs from NPLC-FAIMS-MS experiments (n = 5). The data were generated using 5 consecutive analysis of Splash LipidoMix spiked serum samples.


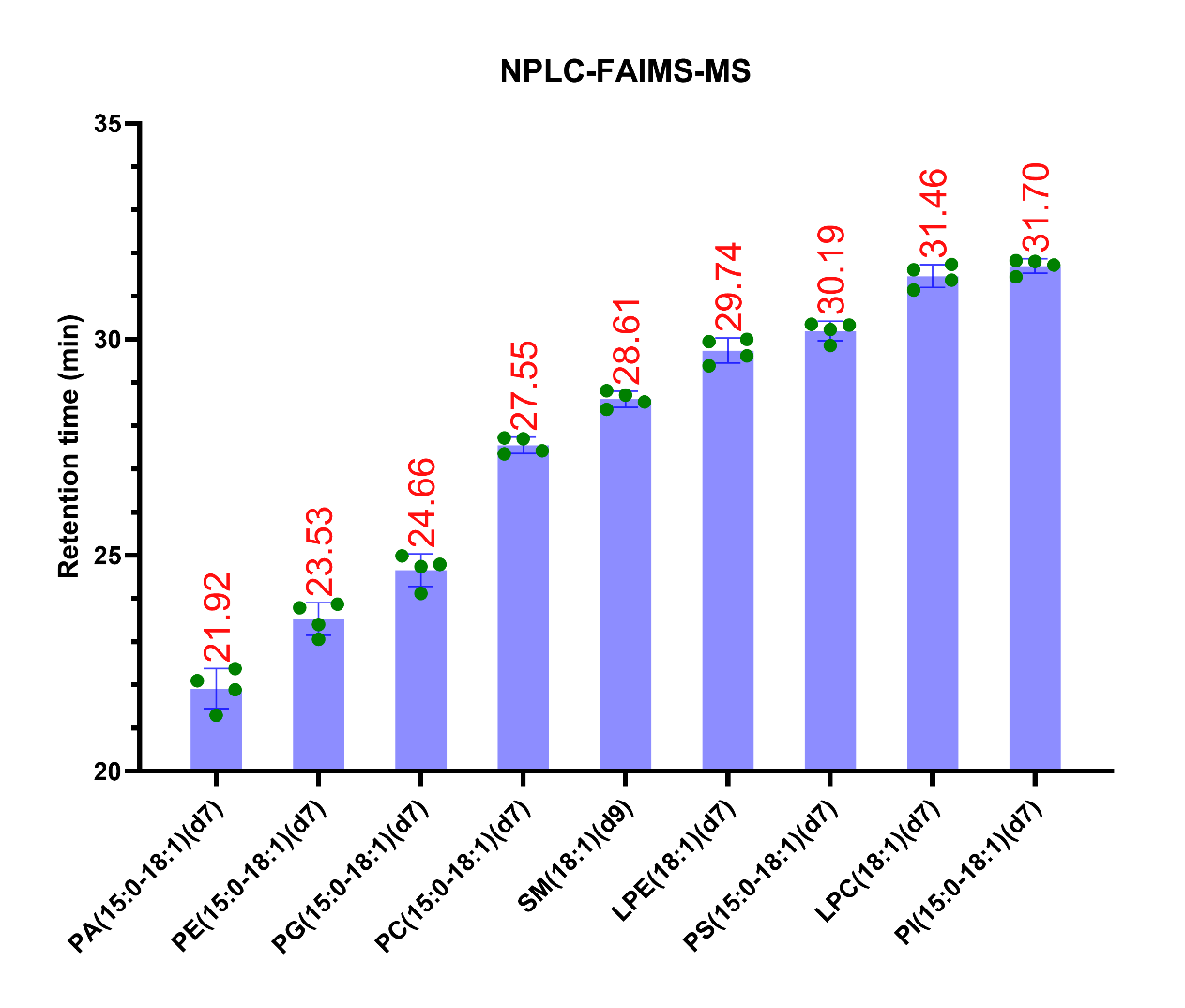


Fig. S3. Retention times of lipid standards in Splash LipidoMix. Data represent mean values from 4 independent NPLC-FAIMS-MS experiments for each isotopic labelled standard.


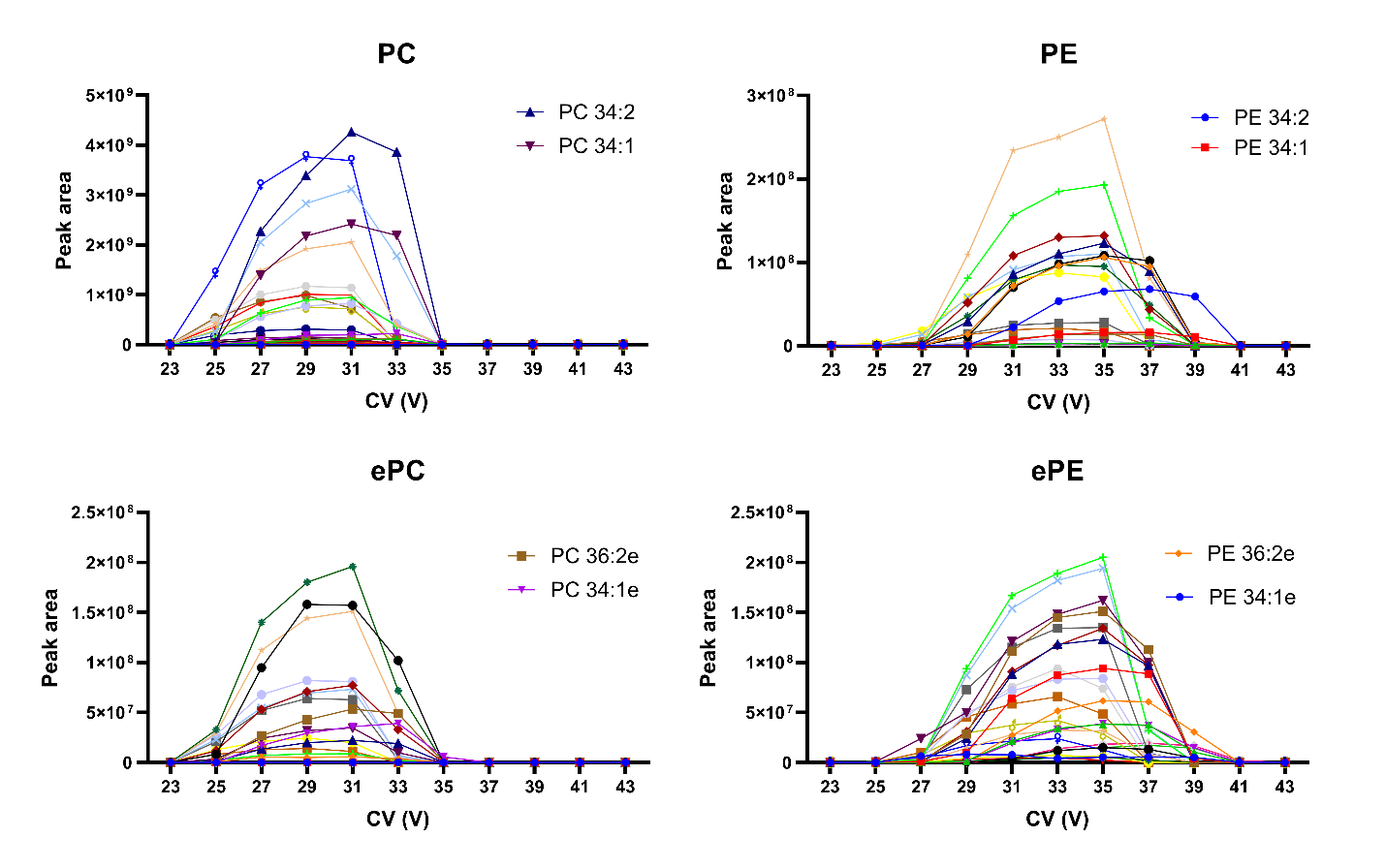


Fig. S4. Peak areas of PC, ePC, PE and ePE at different CV values. NPLC-FAIMS-MS data were acquired under MS1-only mode. The CVs were changed from 23 V to 43 V, with a step of 2 V.


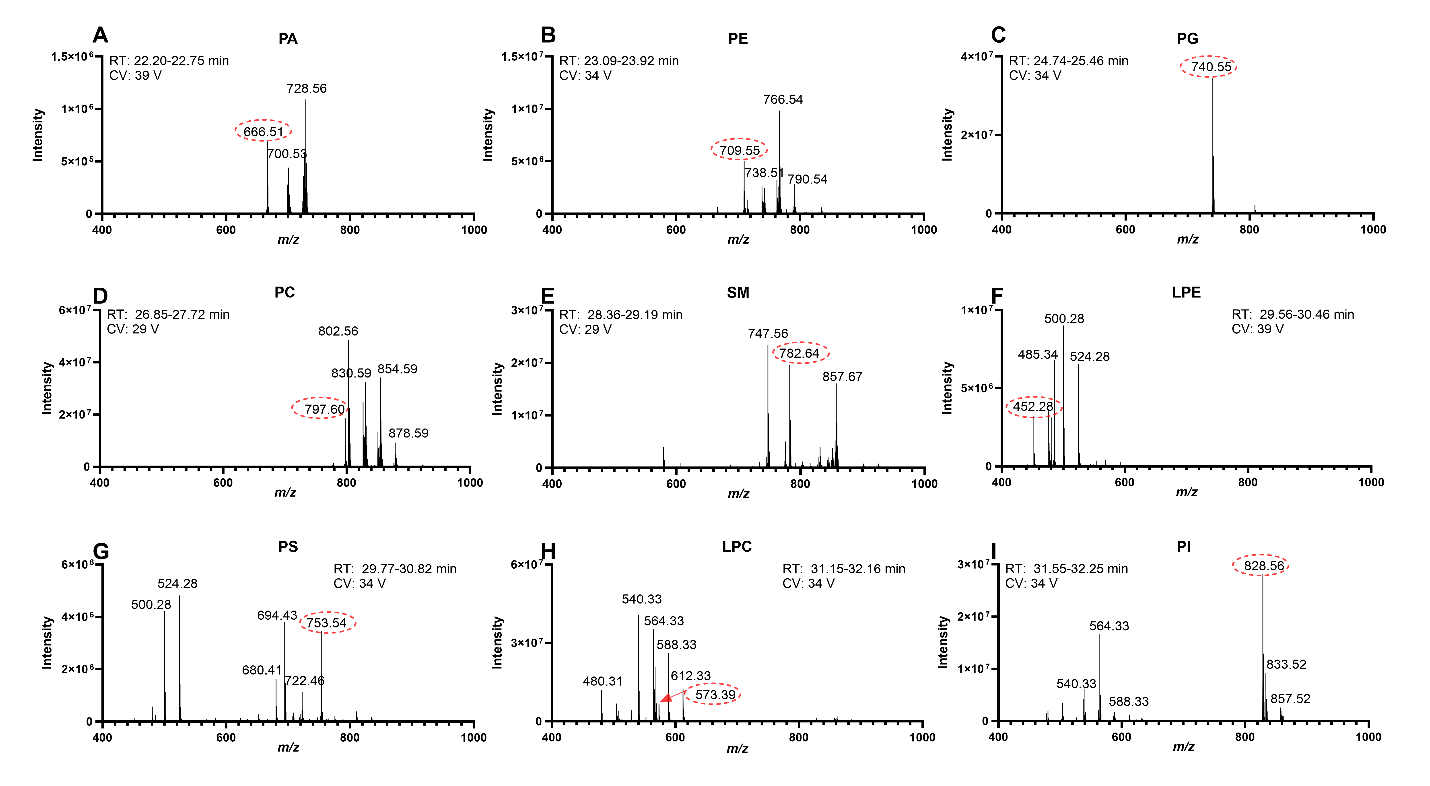
Fig. S5. Extracted MS spectra from NPLC-FAIMS-MS analysis of pooled serum samples. The ions highlighted in red circle correspond to the lipid standards in Splash LipidoMix. (A) PA, CV = 39 V, RT = 22.20 – 22.75 min; (B) PE, CV = 34 V, RT = 23.09 – 23.92 min; (C) PG, CV = 34 V, RT = 24.74 – 25.46 min; (D) PC, CV = 29 V, RT = 26.85 – 27.72 min, (E) SM, CV = 29 V, RT = 28.36 – 29.19; (F) LPE, CV = 39 V, RT = 29.56 – 30.46 min; (G) PS, CV = 34 V, RT = 29.77 – 30.82 min; (H) LPC, CV = 34 V, RT = 31.15 – 32.16 min; (I) PI, CV = 34 V, RT = 31.55 – 32.35 min. The injected quantities of reference standards are 1.1 nmol of PA, 1.1 nmol of PE (15:0-18:1) (d7), 3.8 nmol of PG (15:0-18:1) (d7), 21.3 nmol of PC (15:0-18:1) (d7), 4.2 nmol SM (18:1) (d9), 1.1 nmol of LPE (18:1) (d7), 5 nmol of PS (15:0-18:1) (d7), 4.8 nmol of LPC (18:1) (d7) and 1.1 nmol of PI (15:0-18:1) (d7).


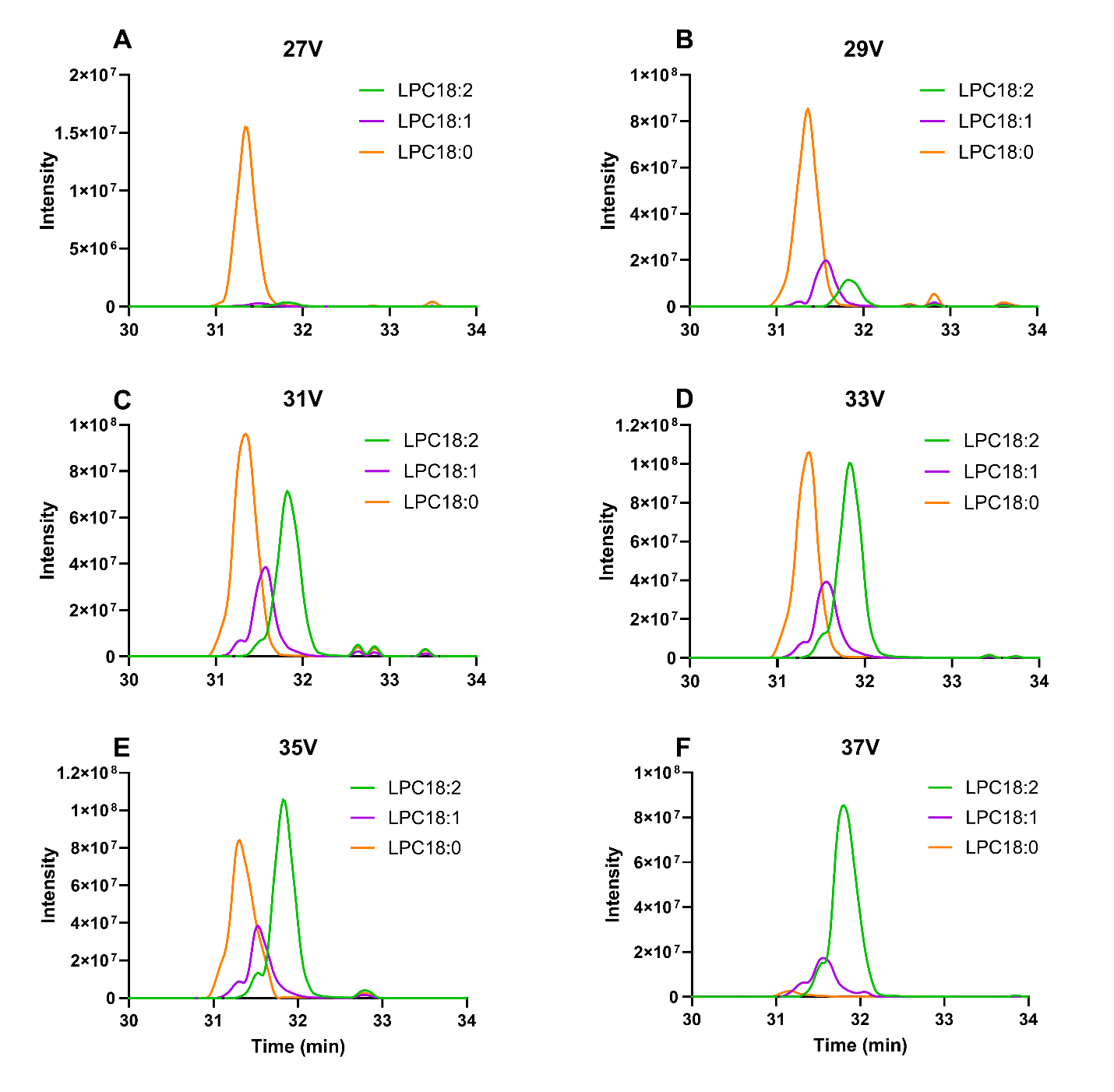


Fig. S6. Extracted ion chromatograms of LPC (18:2), LPC (18:1) and LPC (18:0) at different CV values. (A) CV = 27 V; (B) CV = 29 V; (C) CV = 31 V; (D) CV = 33 V; (E) CV = 35 V; (F) CV = 37 V.


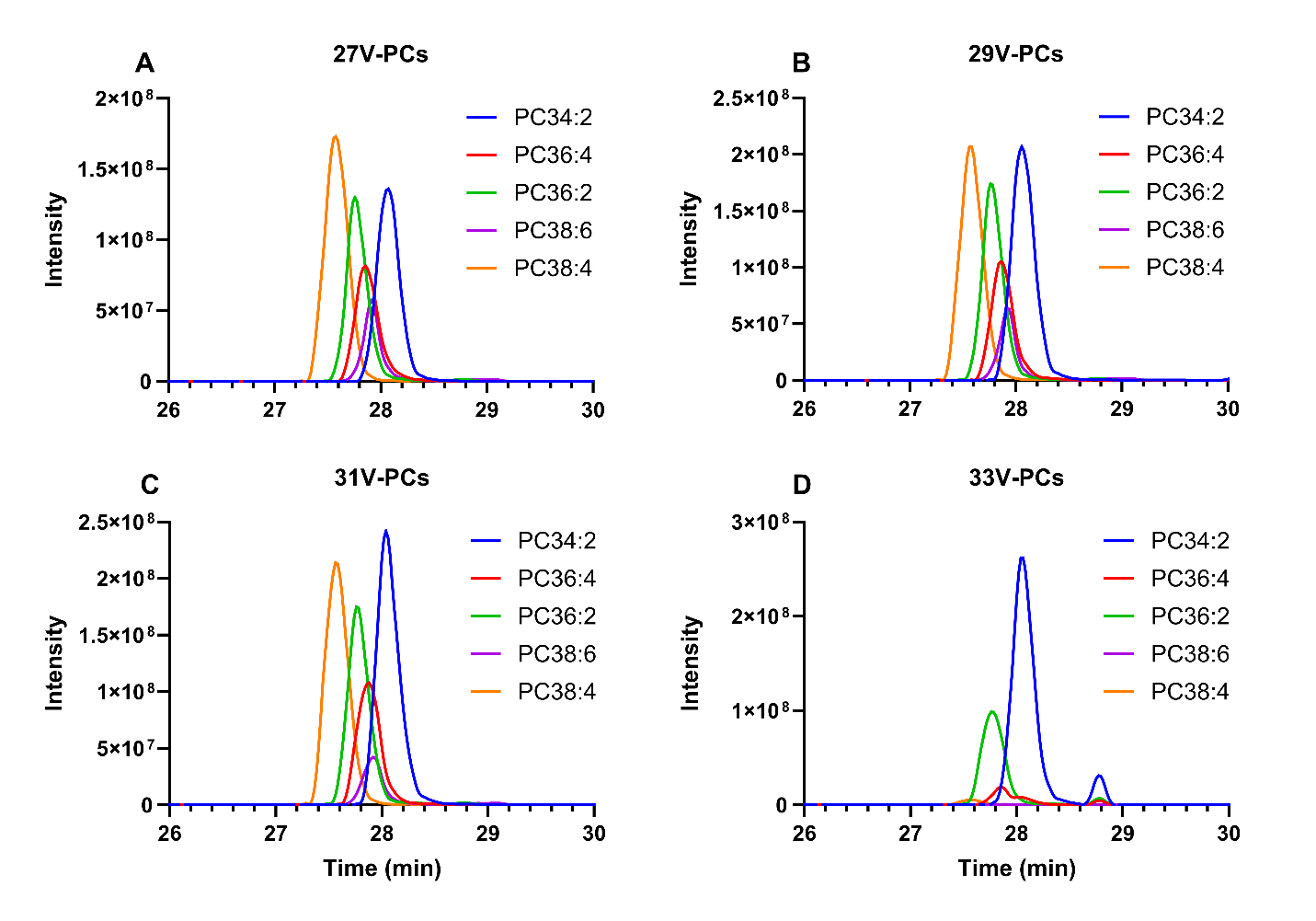


Fig. S7. Extracted ion chromatograms of PC (34:2), PC (36:4), PC (36:2), PC (38:6) and PC (38:4) at different CV values. (A) CV = 27 V; (B) CV = 29 V; (C) CV = 31 V; (D) CV = 33 V.


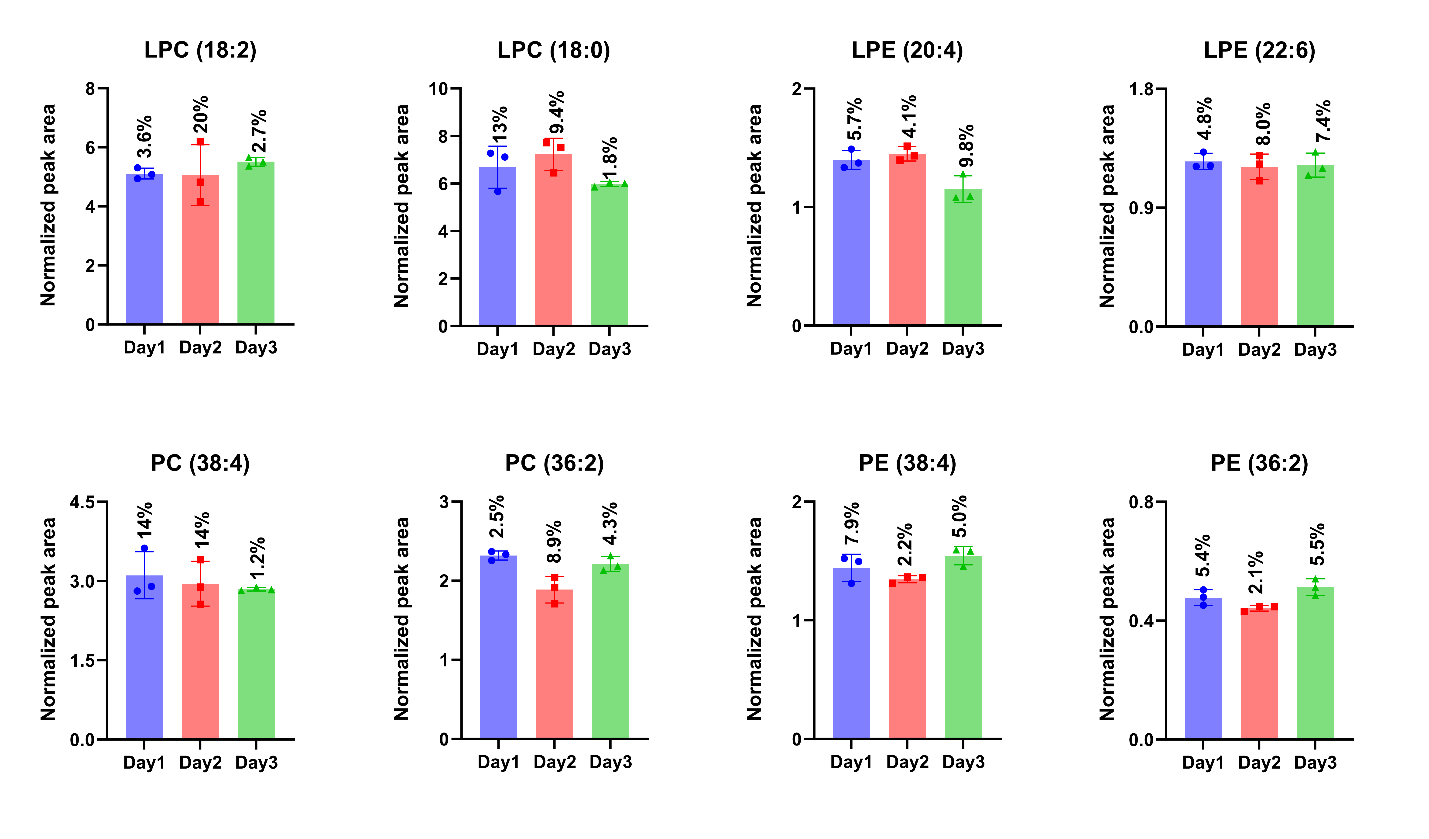


Fig. S8. Intraday reproducibility of normalized peak areas of representative lipids (n = 3). Data were from 3 independent NPLC-FAIMS-MS/MS runs for each day. The peak areas were normalized against spiked reference standards.


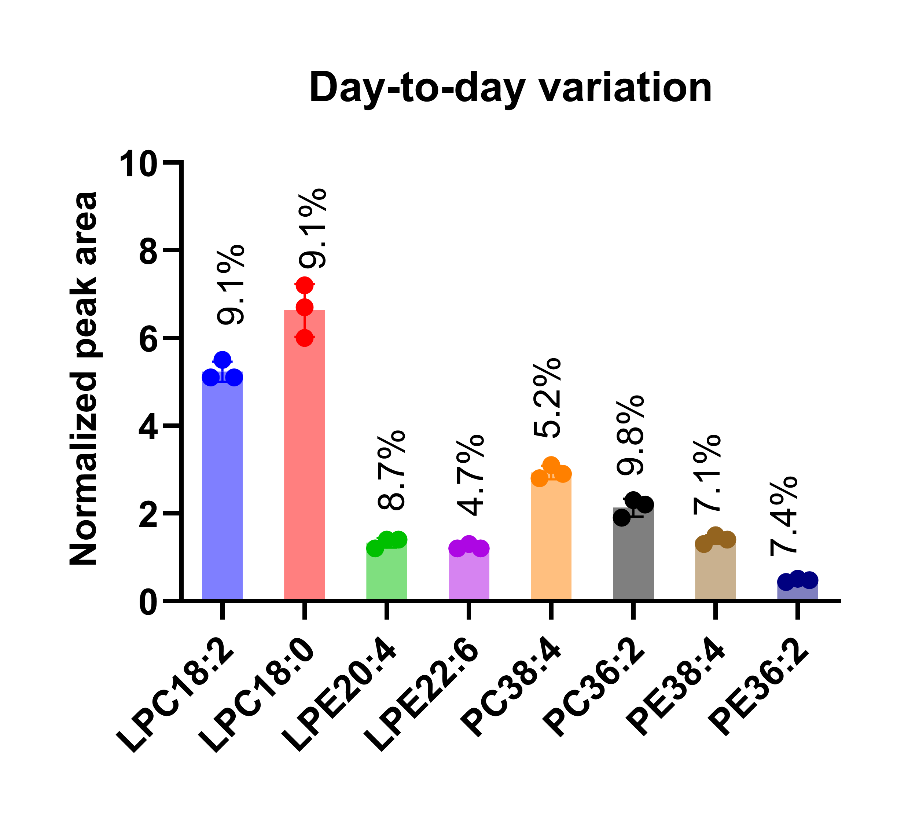


Fig. S9. Day-to-day reproducibility of normalized peak areas of representative lipids from 3 different days (n = 3). The peak areas were normalized against spiked reference standards. The coefficient variation values of the representative species were denoted above the top of their corresponding columns.


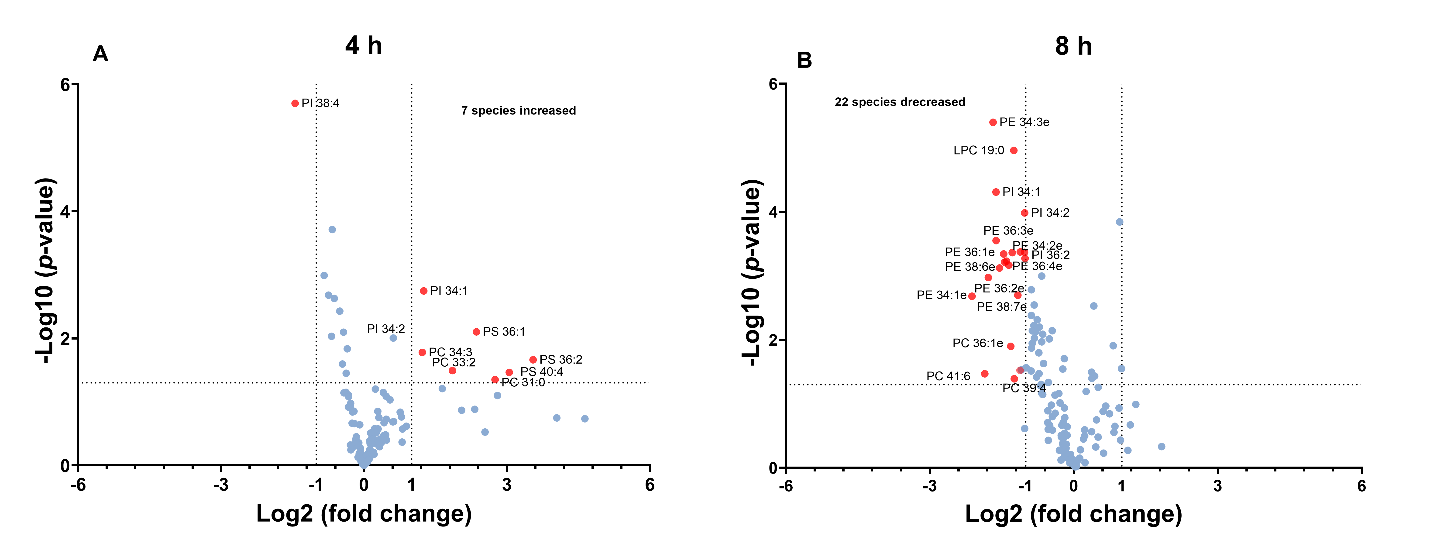


Fig. S10. Volcano plots demonstrating the effect of *A. baumannii* infection on the circulating lipidome from the mouse sera 4 h and 8 h after inoculation. Data were from 3 controls and 5 infected animals, (A) 4 h; (B) 8 h, after inoculation. The red spots highlight those with a fold change ≥ 2 and adjusted p-value ≤ 0.05. Statistical analysis was carried out using multiple unpaired t tests (GraphPad Prism v10.2.3).


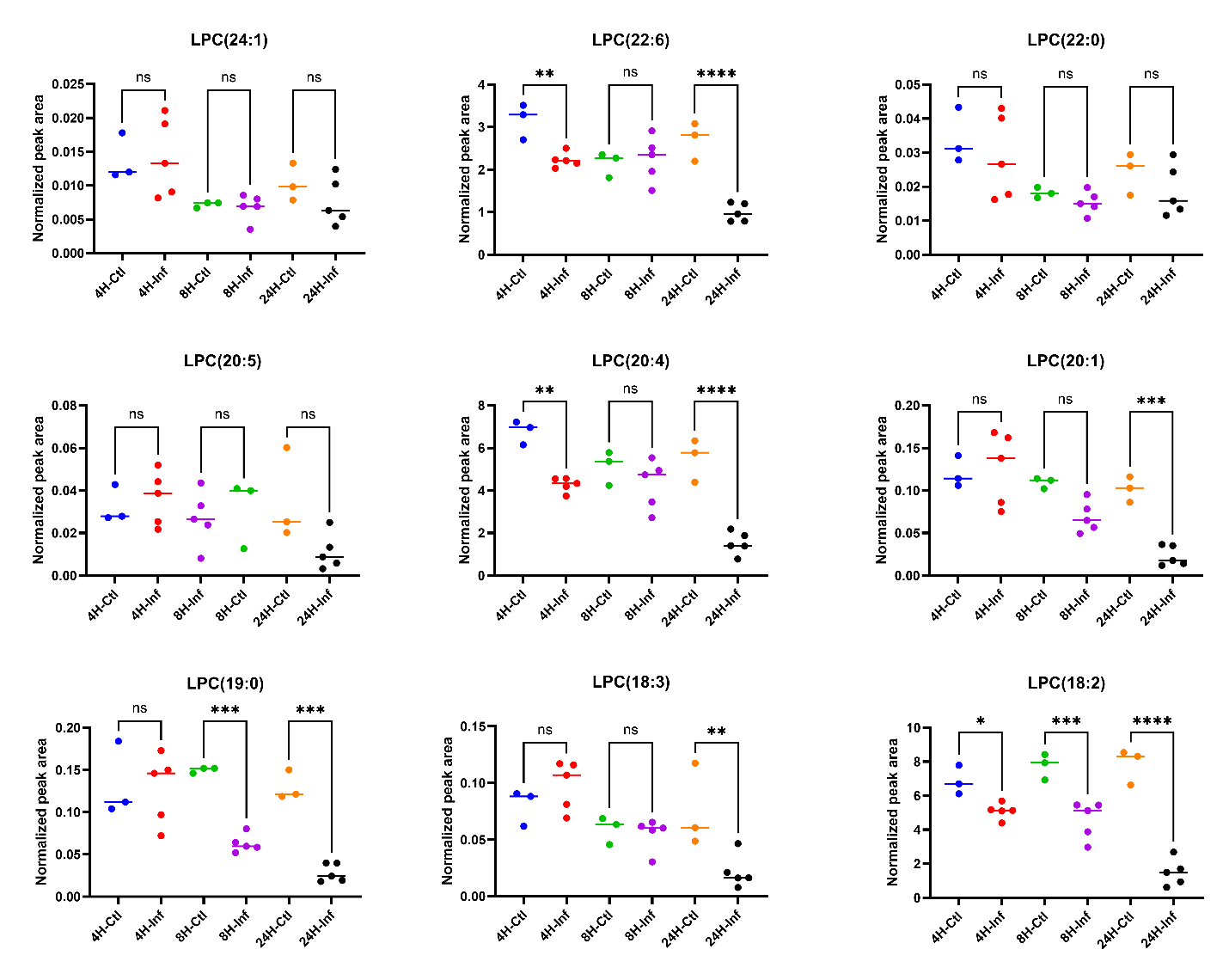


Fig. S11-1. Comparison of relative quantity of LPCs from controls to infection at 4 h, 8 h and 24 h, respectively. The peak areas were normalized against 240 nmol of spiked LPC (18:1) (d7) in 10 µL of mouse serum. Statistical analysis was carried out using a one-way ANOVA (ns = not significant, *P = < 0.05, **P < 0.01, ***P = < 0.001, and ****P = < 0.0001).


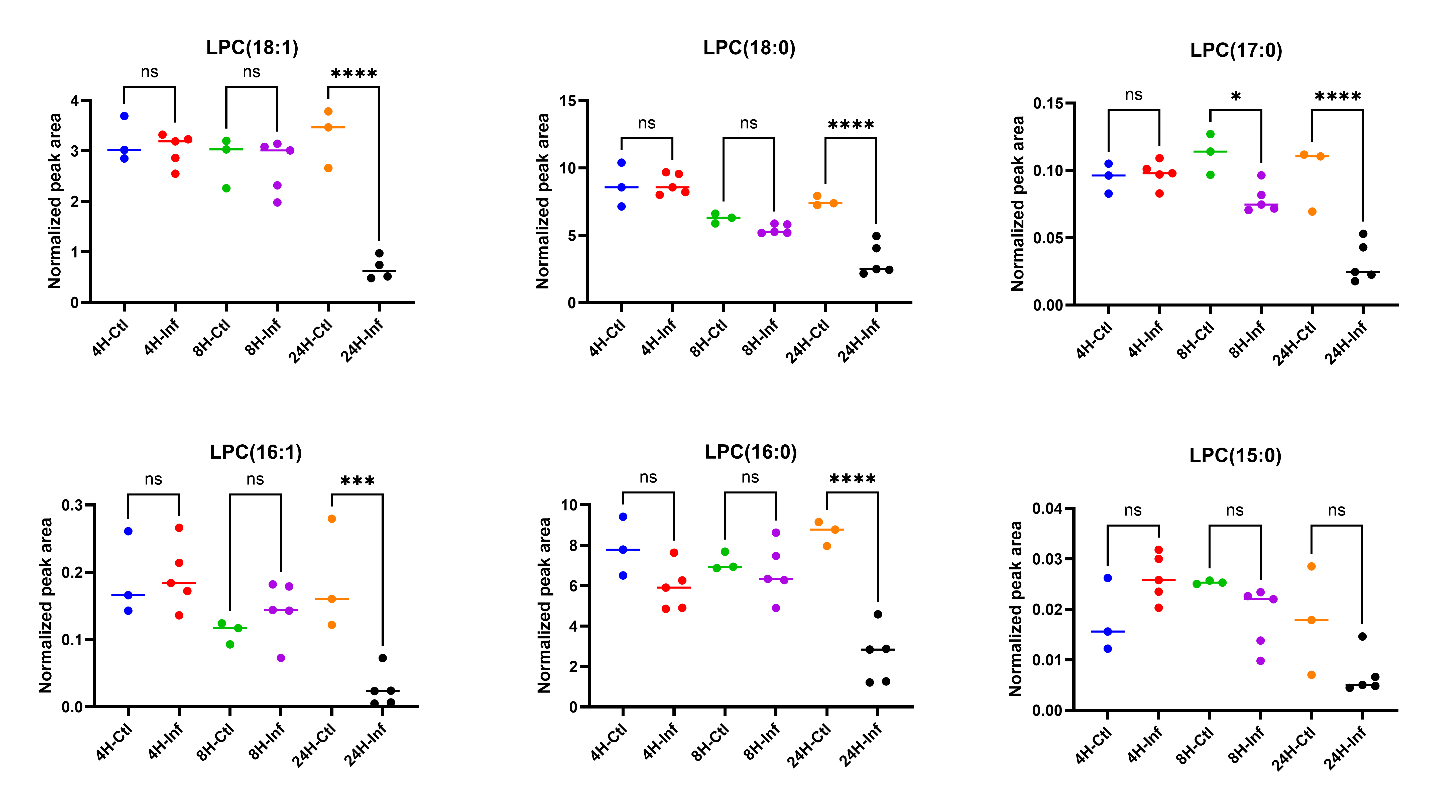


Fig. S11-2. Comparison of relative quantity of LPCs from controls to infection at 4 h, 8 h and 24 h, respectively. The peak areas were normalized against 240 nmol of spiked LPC (18:1) (d7) in 10 µL of mouse serum. Statistical analysis was carried out using a one-way ANOVA (ns = not significant, *P = < 0.05, **P < 0.01, ***P = < 0.001, and ****P = < 0.0001).


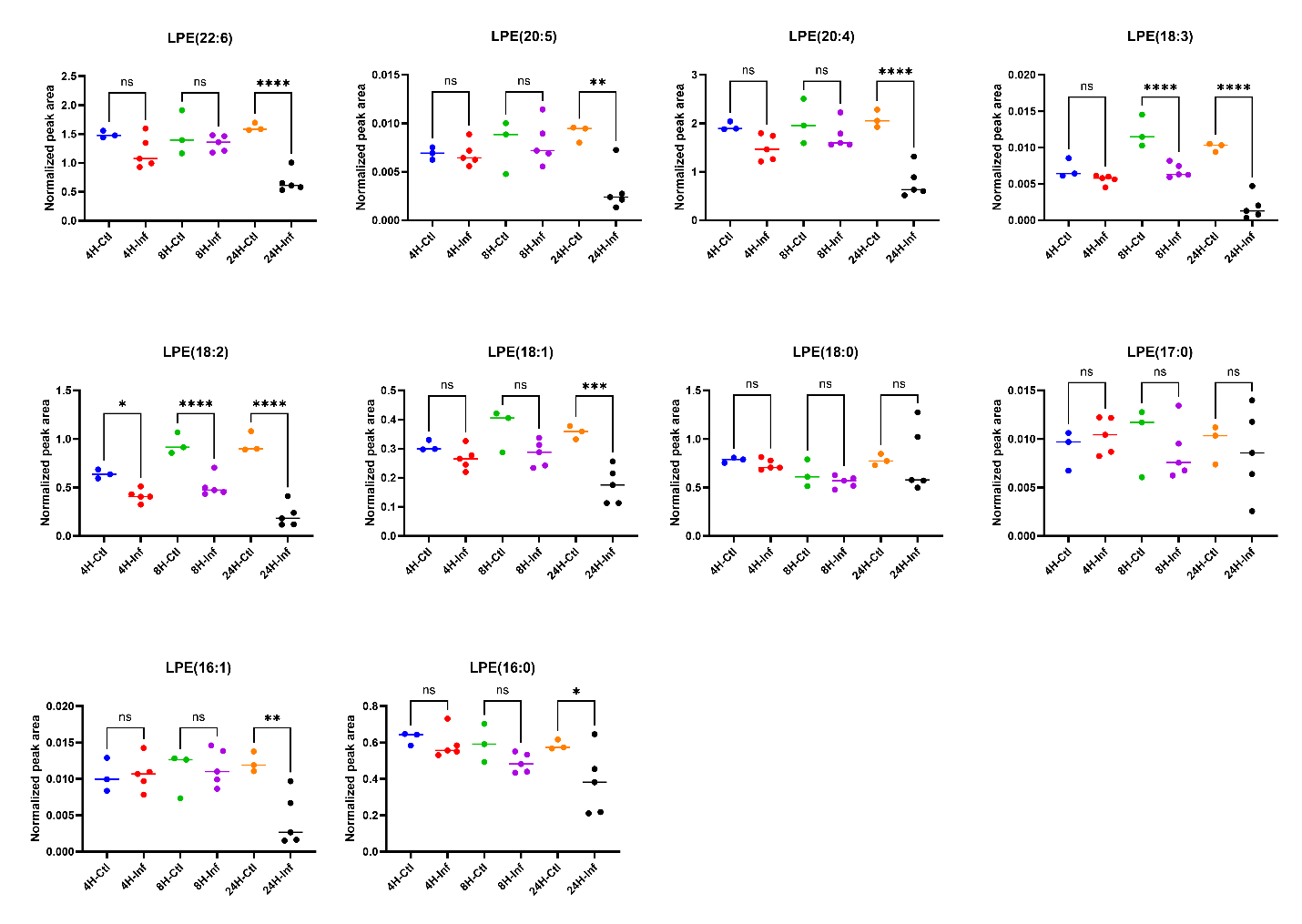


Fig. S12. Comparison of relative quantity of LPEs from controls to infection at 4 h, 8h and 24 h, respectively. The peak areas were normalized against 55 nmol of spiked LPE (18:1) (d7) in 10 µL of mouse serum. Statistical analysis was carried out using a one-way ANOVA (ns = not significant, *P = < 0.05, **P < 0.01, ***P = < 0.001, and ****P = < 0.0001).


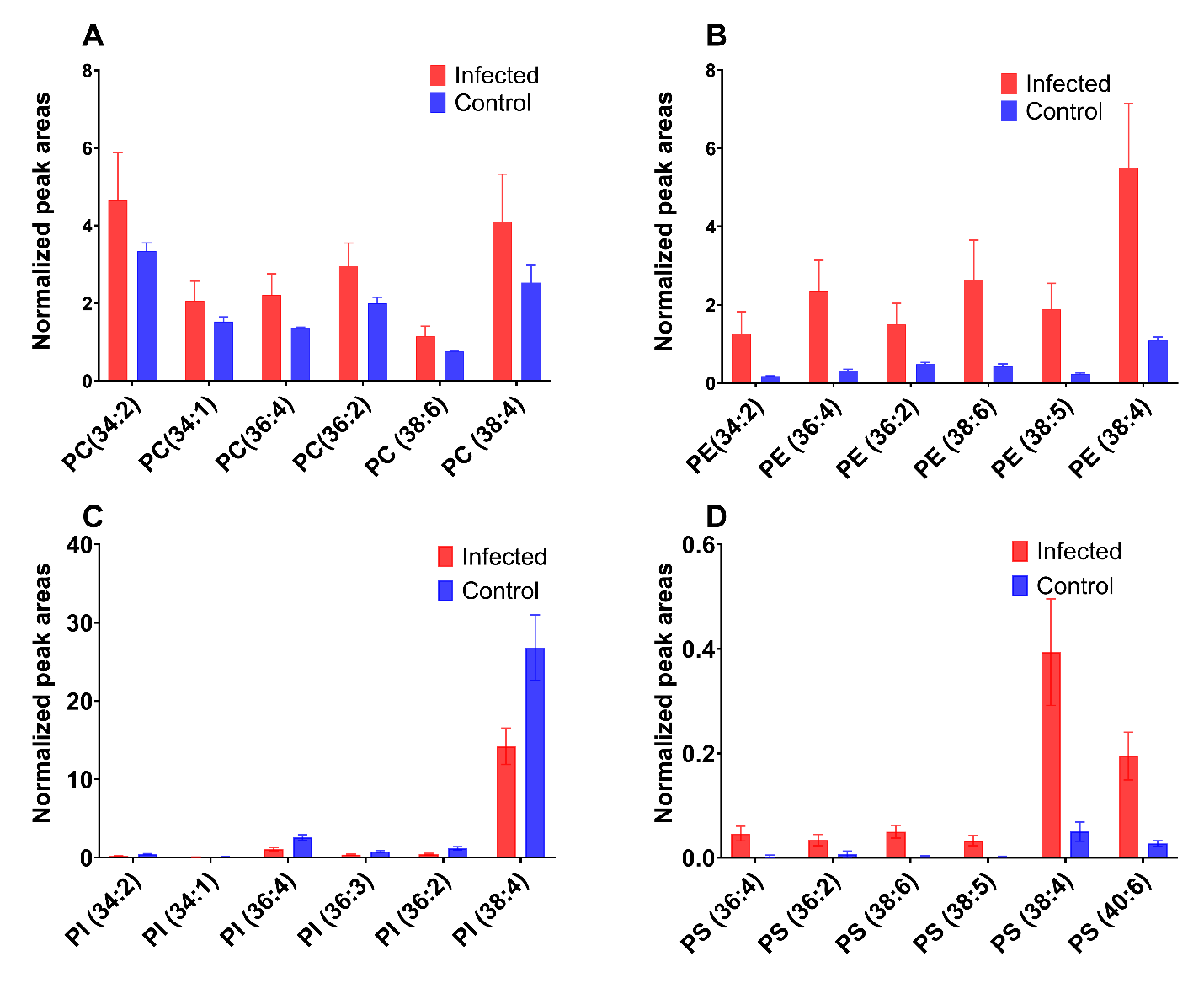


Fig. S13. The impact of *A. baumannii* infection on serum lipid profiles, comparison of relative quantity of lipids between controls and 24 h after inoculation. (A) PCs; (B) PEs; (B) PIs; (C) PSs. The peak areas were normalized against 1065 nmol of spiked PC (15:0-18:1) (d7), 55 nmol of spiked PE (15:0-18:1) (d7), 55 nmol of spiked PI (15:0-18:1) (d7) and 250 nmol of spiked PS (15:0-18:1) (d7) in 10 µL mouse serum for PCs, PEs, PIs and PSs, respectively.


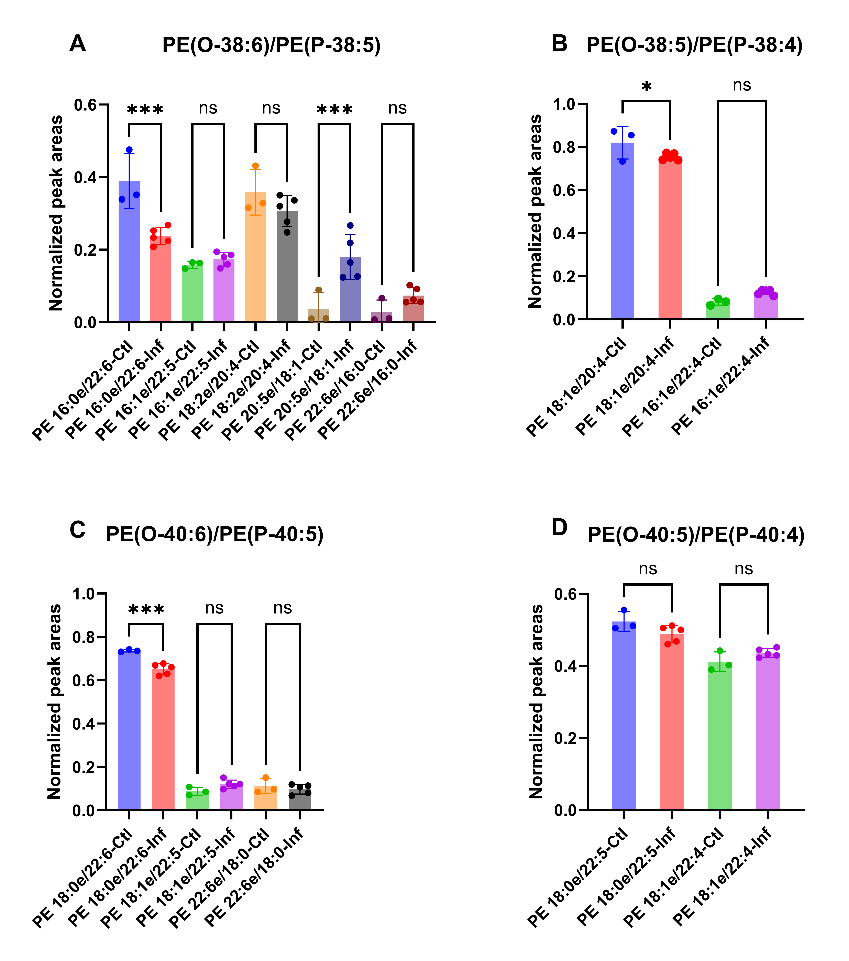


Fig. S14. NPLC-FAIMS-PRM experiment for investigating the effect of *A. baumannii* infection on isobaric PEs, comparison between controls and 24 hours after inoculation, respectively. (A) Normalized peak areas of different compositions of PE(O-38:6)/PE(P-38:5), PRM precursor at *m/z* = 748.5287; (B) Normalized peak areas of different compositions of PE(O-38:5)/PE(P-38:4), PRM precursor at *m/z* = 750.5443; (C) Normalized peak areas of different compositions of PE 4 PE(O-40:6)/PE(P-40:5), PRM precursor at *m/z* = 776.5600; (D) Normalized peak areas of different compositions of PE(O-40:5)/PE(P-40:4), PRM precursor at *m/z* = 778.5756. Peak areas were normalized against the total peak areas of all detected isobaric compositions from same ether PE molecular weight. Statistical analysis was carried out using one-way ANOVA (ns = not significant, *P = < 0.05, **P < 0.01, ***P = < 0.001, and ****P = < 0.0001).
